# The Causal Evidence of Birth Weight and Female-Related Traits and Diseases: A Two-Sample Mendelian Randomization Analysis

**DOI:** 10.3389/fgene.2022.850892

**Published:** 2022-08-12

**Authors:** Renke He, Rui Liu, Haiyan Wu, Jiaen Yu, Zhaoying Jiang, Hefeng Huang

**Affiliations:** ^1^ International Institutes of Medicine, The Fourth Affiliated Hospital, Zhejiang University School of Medicine, Yiwu, China; ^2^ Department of Reproductive Endocrinology, Women’s Hospital, School of Medicine, Zhejiang University, Hangzhou, China; ^3^ Key Laboratory of Reproductive Genetics, Ministry of Education, School of Medicine, Zhejiang University, Hangzhou, China; ^4^ Shanghai Frontiers Science Center of Reproduction and Development, Shanghai, China; ^5^ Research Units of Embryo Original Diseases, Chinese Academy of Medical Sciences, Shanghai, China

**Keywords:** birthweight, reproductive hormones, body mass index, menarche, leiomyoma, Mendelian randomization

## Abstract

**Objectives** **:** A large meta-analysis indicated a more pronounced association between lower birth weight (BW) and diseases in women but less concern about the causality between BW and female-related phenotypes and diseases.

**Methods:** Mendelian randomization (MR) analysis was used to estimate the causal relationship between two traits or diseases using summary datasets from genome-wide association studies. Exposure instrumental variables are variants that are strongly associated with traits and are tested using four different statistical methods, including the inverse variance weighting, MR-Egger, weighted median, and weighted mode in MR analysis. Next, sensitivity analysis and horizontal pleiotropy were assessed using leave-one-out and MR-PRESSO packages.

**Results:** The body mass index (BMI) in adulthood was determined by BW (corrected β = 0.071, *p* = 3.19E-03). Lower BW could decrease the adult sex hormone-binding globulin (SHBG) level (β = −0.081, *p* = 2.08E-06), but it resulted in increased levels of bioavailable testosterone (bio-T) (β = 0.105, *p* = 1.25E-05). A potential inverse effect was observed between BW and menarche (corrected β = −0.048, *p* = 4.75E-03), and no causal association was confirmed between BW and the risk of endometriosis, leiomyoma, and polycystic ovary syndrome.

**Conclusion:** Our results suggest that BW may play an important role and demonstrates a significant direct influence on female BMI, SHBG and bio-T levels, and menarche.

## Introduction

The hypothesis of “developmental origins of adult disease” was first stated by Barker (Barker and Osmond, 1986) in the 20th century, which mainly explained that the adverse influences in the early developmental period could cause permanent changes in physiology and metabolism, which finally results in an increased risk of disease in adulthood. Thus, birth weight (BW) is widely used as an indicator of exposure during the intrauterine period and early life development (Peck et al., 2003; Scharf et al., 2016). Numerous observational studies provided evidence for the correlation between reduced BW and increased risk of adult diseases, such as type 2 diabetes mellitus (T2DM) (Carlsson et al., 1999; Whincup et al., 2008), coronary heart disease (CHD) (Ferrie et al., 2006; Morley et al., 2006), hypertension (Eriksson et al., 2000a; Tamakoshi et al., 2006), and stroke (Eriksson et al., 2000b). In particular, it is worth noting that only women demonstrated an increased risk of T2DM and CHD with a raised BW in a recent sex-specific binary meta-analysis (Knop et al., 2018), indicating that BW is more acceptable in predicting the correlation between several traits and diseases in women. Indeed, early observational studies provided controversial evidence supporting the association between BW and female-related traits, including female-only body mass index (BMI) (Zhao et al., 2012; Jelenkovic et al., 2017), reproductive hormones (estradiol [E_2_] (Jasienska et al., 2006; Espetvedt Finstad et al., 2009), testosterone (Ruder et al., 2011), anti-Mullerian hormone [AMH] (Dior et al., 2021)), menarche (Juul et al., 2017; Fan et al., 2018), menopause (Tom et al., 2010; Bjelland et al., 2020), and female-specific diseases (polycystic ovaries syndrome [PCOS] (Cresswell et al., 1997), endometriosis (Olšarová and Mishra, 2020), and leiomyomata (Wise et al., 2012)), which can influence women’s reproductive health and life expectancy. However, whether the identified correlation between BW and these female-related traits represents a truly causal relationship remains uncertain because of bias, pleiotropy, or common confounders during prenatal life (Ruiz-Narváez et al., 2014; Kahn et al., 2017; Lawlor et al., 2017).

Two-sample Mendelian randomization (TSMR), a novel and popular analysis tool, was used to estimate the causal inference in observational studies, which avoided all possible and potential biases from confounding factors. The fundamental theory of TSMR is that during the period when gametes were formatted and combined, the alleles of genetic variants were segregated randomly based on Mendel’s law, which led to their independence with confounding factors such as the environment, age, and sex. To some extent, this implies that the TSMR results are stable and convincing.

In recent years, Mendelian randomization (MR) studies provided evidence of a positive association between lower birth weight (LBW) and T2DM (Huang et al., 2019) and stroke (Wang et al., 2020), a negative association with chronic kidney disease (Yu et al., 2020), and no relationship with asthma (Zeng et al., 2019). Furthermore, no related or specific reports focused on women’s health and diseases exist. Here, a large TSMR analysis was conducted to comprehensively estimate the causality of BW on eight related traits and three common reproductive endocrine diseases in adulthood. Our results remained statistically significant and robust after validating the heterogeneity, sensitivity, and horizontal pleiotropy.

## Materials and Methods

### Data Sources

First, a genome-wide association studies (GWAS) of female BMI was obtained from a large genome-wide meta-analysis combining summary data from the United Kingdom Biobank and the GIANT consortium (European ancestry, *n* = 143,677) (Pulit et al., 2019). The summary datasets of reproductive hormones in women were identified from the United Kingdom Biobank (European ancestry, total testosterone (TT) level, *n* = 230,454; bioavailable testosterone (bio-T), *n* = 188,507; sex hormone-binding globulin (SHBG) level, *n* = 189,473; E_2_ level, *n* = 163,985) (Ruth et al., 2020; Schmitz et al., 2021). The summarized statistics for the AMH was collected from a genome-wide meta-analysis including five cohorts and 3,344 premenopausal women (Ruth et al., 2019). In addition, other traits closely related to the female sex were age at menarche (AAM) and menopause, which were sourced from the MER-IEU Consortium and included 243,944 and 211,114 women, respectively. To conclude, we decided on three common female-specific reproductive endocrine diseases—endometriosis, leiomyoma, and PCOS—to evaluate their causal relationship. The GWAS outcome of endometriosis and leiomyoma was obtained from the FinnGen biobank, recruiting 6,502 cases and 57,407 controls and 14,569 cases and 72,789 controls, respectively. The population of PCOS patients was determined through a large-scale meta-analysis, including six studies and 24,267 samples (Day et al., 2018). The detailed information and characteristics of the GWAS outcomes are listed in Supplementary Table S1.

### Selection of Instrumental Variables

First, the plinked version of 47 independent single-nucleotide polymorphisms (SNPs) were identified as instrumental variables (IVs) representing interest exposure (e.g., BW) to perform MR analysis, which showed a strong association with statistical significance (*p* < 5.0E-08) based on early growth genetics (EGG) consortium research (Zeng and Zhou, 2019) (Table 1). Up to now, the EGG consortium study is the largest GWAS on BW (a continuous trait) and contains 16,245,523 imputed SNPs based on 153,781 infants collected from more than 30 studies (Supplementary Table S2). Another different version of the 48 SNPs was used to validate the robustness of the results (Horikoshi et al., 2016) (Supplementary Table S3). Then, all IVs were independent after performing the clumping procedure (*R* ^2^ = 0.001, kb = 10,000) and removing the linkage disequilibrium between SNPs. Third, the F-statistics has been applied to ensure the sufficient power of IVs in the MR analysis, and the results proved strong effect sizes with overall F-statistics > 10. The SNP of IVs for lower BW was presented by supplying a negative sign on the estimated BW effect (Zeng and Zhou, 2019). To conclude, all the above procedures were run in the R software (version 4.0.3) using the “TwoSampleMR” package to automatically prune SNPs with linkage dependence.

### Multivariable Mendelian Randomization

The inverse variance weighting method was applied in the two-sample multivariable MR (MVMR), which fits multiple risk factors as exposures (e.g., fetal body weight and BMI in our study), to simultaneously estimate their genetically predicted effects on an outcome (e.g., concentration of SHBG and bio-T, menarche). This analysis allowed us to estimate the direct effect of LBW (i.e., the effect after accounting for adult BMI) and its indirect effect (i.e., the effect mediated by BMI in adulthood) on each female trait. To evaluate the causal effects of BMI-adjusted LBW in our study, MVMR analysis was performed, which included SNPs that reached genome-wide significance (*p* < 5.00E-8) in both GWAS of LBW and BMI. For these two exposures, we used nonoverlapping populations. After excluding SNPs with a pairwise *R* ^2^ > 0.001, 966 independent SNPs were used as IVs in the analysis. Then, MVMR analysis was conducted using both the MVMR and TSMR packages in the R software.

### Statistical Analysis

Two-sample MR was applied to the GWAS data in our study. We chose the IVW random-effects model as the main tool to estimate causal associations based on GWAS data for BW and female-related traits and diseases. Next, we performed an estimation using three other methods—MR-Egger (MRE), weighted median (WM), and weighted mode—ensuring the stability and reliability of the results. Also, we measured the causal effect heterogeneity using Cochran’s Q test and I^2^ statistics, and the “leave-one-out” sensitivity analysis was performed to ascertain whether the heterogeneity was caused by specific SNPs. The MRE and Mendelian Randomization Pleiotropy RESidual Sum and Outlier (MR-PRESSO) analysis (Verbanck et al., 2018) were conducted to eliminate the bias caused by horizontal pleiotropy, outlier SNPs were identified using the MR-PRESSO analysis, and the results were corrected. Further, all results are presented in forest plots, scatterplots, leave-one-out plots, and funnel plots. All procedures were repeated using another version of the exposure SNPs. In general, *p*-values < 0.05 were considered statistically significant, but in multiple testing, the *p*-value threshold was adjusted through Bonferroni correction (*p* < 0.05/11 = 4.55E-03). If the outcomes are continuous variables, the estimated effects are exhibited as a beta effect (β), standard error (se), and *p*-value. They are presented as odds ratios (Ors) with 95% confidence intervals (Cis). Also, the R software and “TwoSampleMR” package were used for all analyses (Yavorska and Burgess, 2017).

## Results

### Higher Birth Weight May Determine Higher Body Mass Index in Women

**FIGURE 1:**
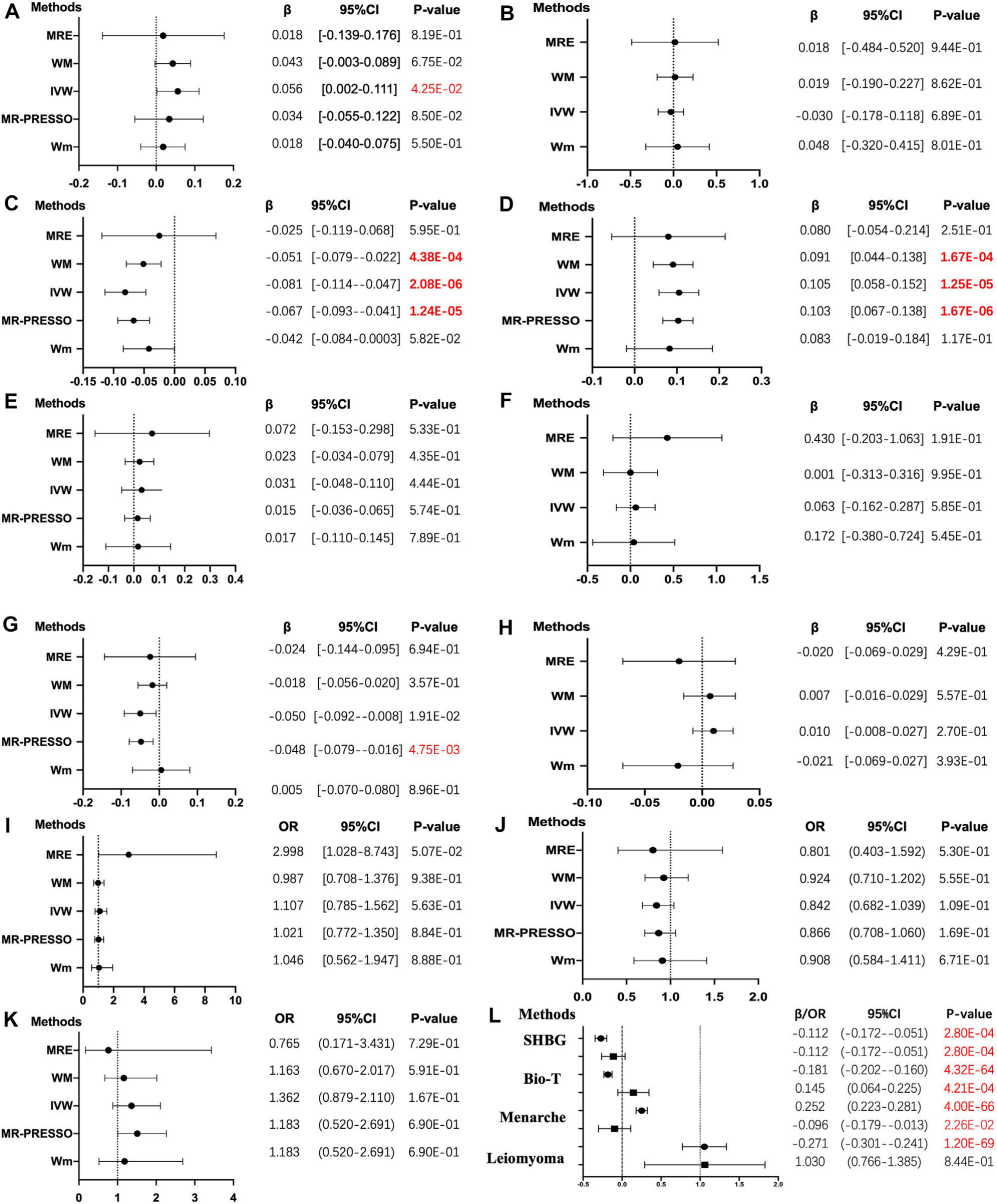
The results of four different methods of Mendelian randomization (MR) analysis. (The MR analysis showing the effect of the exposure SNPs on the outcomes. **(A–K)**: **(A)** body mass index, BMI; **(B)** estradiol, E_2_; **(C)** sex hormone-binding globulin, SHBG; **(D)** bioavailable testosterone, bio-T; **(E)** total testosterone, TT; **(F)** anti-Mullerian hormone, AMH; **(G)** menarche; **(H)** menopause; **(I)** endometriosis; **(J)** leiomyoma; **(K)** polycystic ovarian syndrome, PCOS. **(L)** in the multivariable MR analysis, each trait with two results is presented. The solid dot means the causal effects of LBW on traits, whereas the square means BMI on traits in the MVMR. MRE, MR-Egger; WM, weighted median; Wm, weighted mode; IVW, inverse variance weighting; MR-PRESSO, MR-Egger and Mendelian Randomization Pleiotropy RESidual Sum and Outlier. The results of the continuous outcomes are presented by β [95% CI], whereas the binary outcomes are shown by OR [95% CI]. Numbers in red mean *p-*values < 5.00E-02 and red and bold font means *p-*values < 4.55E-03)

The primary 47-SNP IVW analysis provided suggestive evidence for a positive causal relationship between BW and BMI (β = 0.056, *p* = 4.25E-02). In addition, similar but more significant results were identified in the 48-SNP IVW analysis (β = 0.071, *p* = 7.63E-03) (Figure 1, Supplementary Figure S1).

### The Causality Between Lower Birth Weight and Reproductive Hormones

As for reproductive hormones, lower BW demonstrated a positive effect on bio-T levels (β = 0.105, *p* = 1.25E-05) in the 47-SNP version and the same causality in the validated 48-SNP version (β = 0.103, *p* = 1.42E-04), but it demonstrated an inverse effect on SHBG concentration (β = −0.081, *p* = 2.08E-06 versus β = −0.075, *p* = 9.36E-05). However, no evidence showed an association between lower BW and levels of E_2_ (β = −0.030, *p* = 6.89E-01), TT (β = 0.031, *p* = 0.444), and AMH (β = 0.063, *p* = 5.85E-01) (Figure 1, Supplementary Figure S1).

### Lower Birth Weight May Result in Higher Risk of Early Age at Menarche

The results of the IVW analyses showed that lower BW tended to exhibit a negative causal effect on AAM, but it did not reach the corrected *p*-value of strong significance (β = −0.048, *p* = 1.90E-02 versus β = −0.053, *p* = 9.82E-03), whereas no relationship was observed between LBW and age at natural menopause (ANM) (β = 0.010, *p* = 2.70E-01) (Figure 1, Supplementary Figure S1).

### No Association Was Identified Between Lower Birth Weight and Three Reproductive Endocrine Diseases

No evidence of causal effects was found between a unit lower BW and endometriosis, leiomyoma, and PCOS, even after the heterogeneity and horizontal pleiotropy were eliminated (OR = 1.107; 95% CI = [0.785–1.562], *p* = 5.63E-01; OR = 0.842; 95% CI = [0.682–1.039], *p* = 109E-01; OR = 1.362; 95% CI = [0.879–2.110], *p* = 1.67E-01). However, 48 LBW SNPs showed potential causality with leiomyoma (OR = 0.791; 95% CI = [0.629–0.994], *p* = 4.46E-02) (Figure 1, Supplementary Figure S1).

### Multivariable Mendelian Randomization

Applying MVMR resulted in the majority of effect estimates identified in the previous analysis being strengthened to include the adjustment for adult BMI. In the MVMR analysis controlling for BMI, more robust evidence was found for a direct and negative causal effect of LBW on SHBG concentration (β = −0.112, 95% CI = [−0.172–0.051]) and AAM (β = −0.096, 95% CI = [−0.179–0.013]) and a positive effect of LBW on bio-T levels (β = 0.145, 95% CI = [0.064–0.225]). Moreover, the weak relationship between LBW and leiomyoma was eliminated in MVMR (OR = 1.030, 95% CI = [0.766–1.385]). The causal relationships estimated from MVMR (including LBW and BMI) were consistent with the univariable IVW analysis (LBW) for SHBG, bio-T, and menarche, except for leiomyoma (Supplementary Table S6, Figure 1L).

### Sensitivity Analysis

**FIGURE 2:**
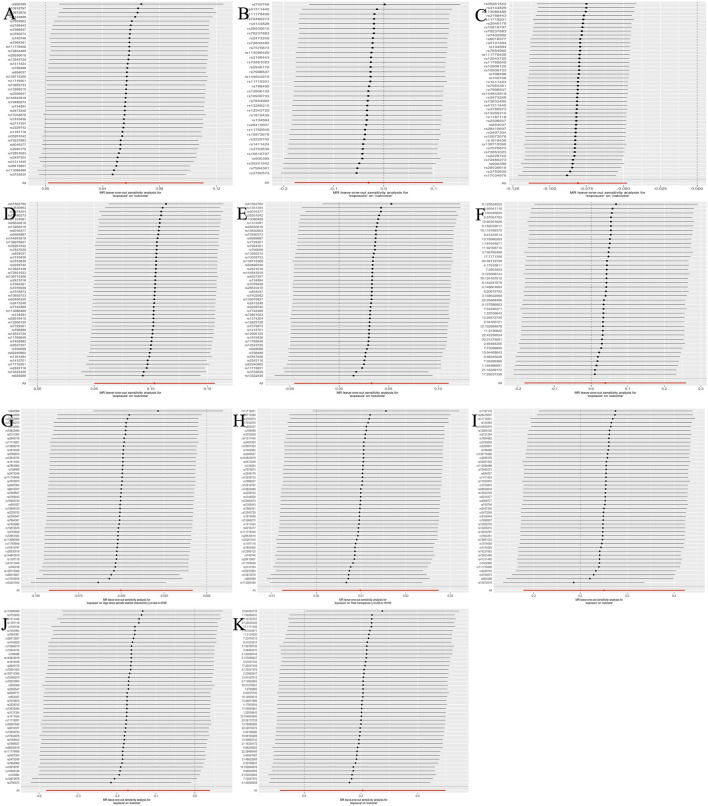
The leave-one-out analysis plot (The estimation effects are reported per SD increase in the exposure, and error bars represent 95% confidence intervals).

**FIGURE 3:**
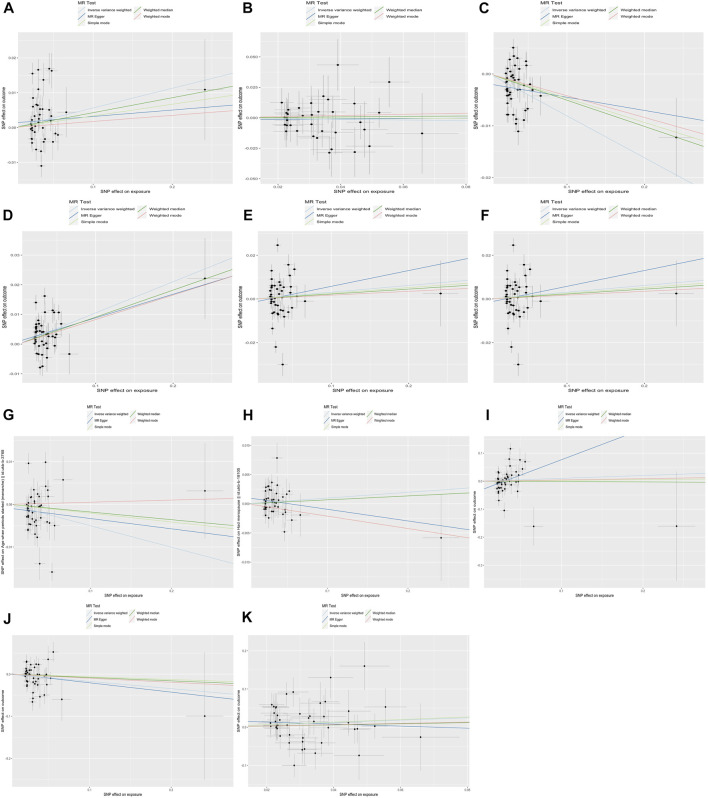
The results of the scatter plot.

**FIGURE 4:**
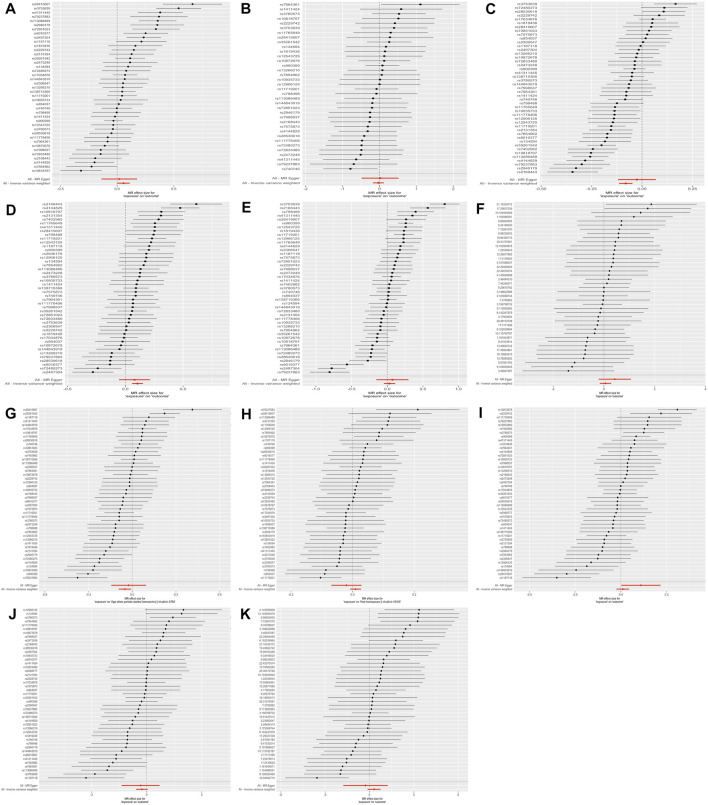
The results of the forest plot.

**FIGURE 5:**
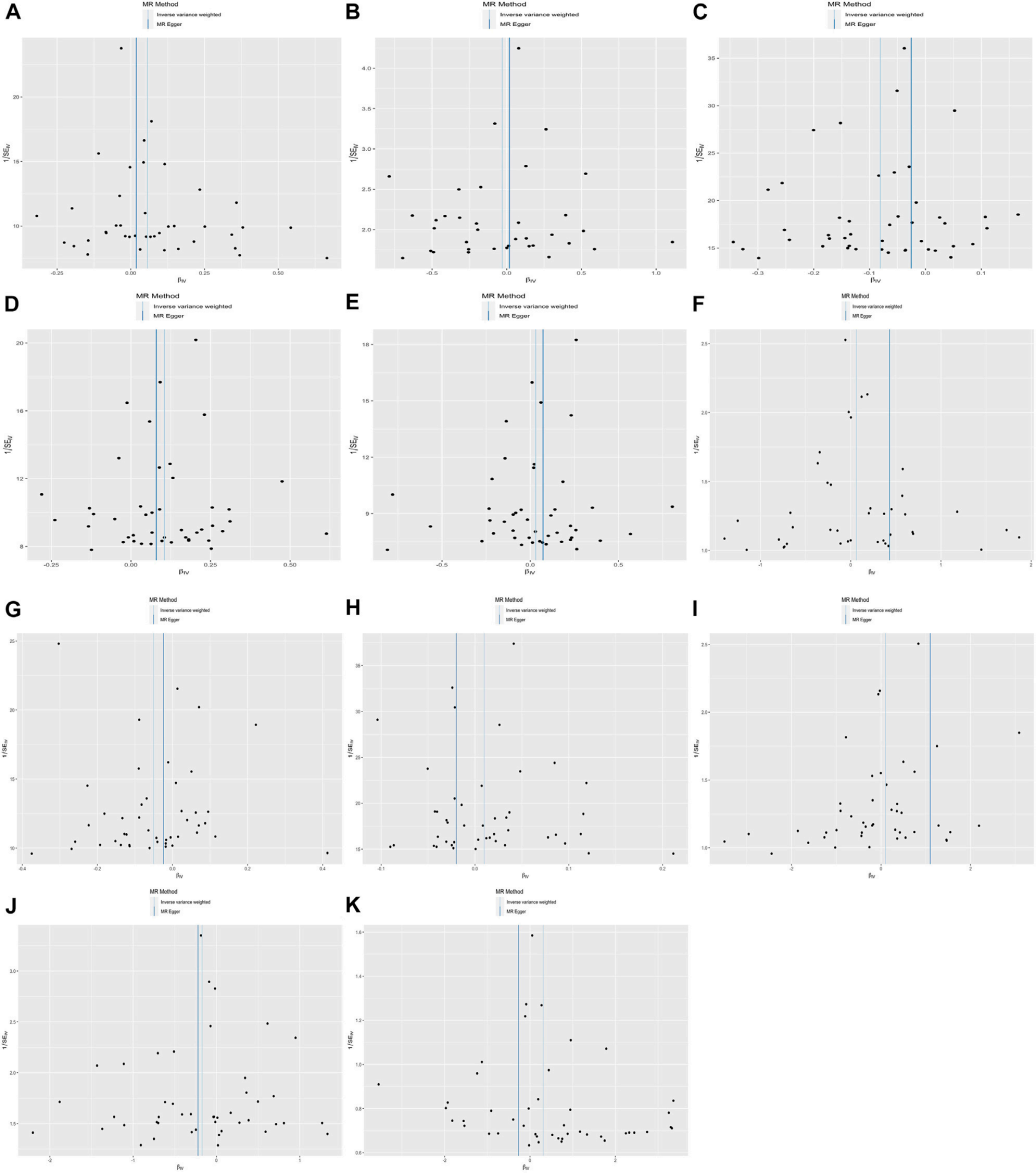
The results of the funnel plot.

Also, the measurement of WM was used to test sensitivity. Similar results proved the negative association between lower BW and SHBG (β= −0.051, *p* = 4.38E-04) and positive causality with bio-T (β= 0.091, *p* = 1.67E-04). Next, conversely, the correlation between BW and BMI was not consistent with our previous findings. All results of the MRE intercept were close to zero and *p* > 0.05, which suggested no horizontal pleiotropy. Owing to the existing heterogeneity, a leave-one-out analysis was applied and presented in the plots (Figure 2, Supplementary Figure S2). Next, the horizontal line and black points in the leave-one-out plot of TT, E_2_, AMH, menopause, endometriosis, leiomyoma, and PCOS crossed the zero line, suggesting potential heterogeneity. The scatterplot, forest plot, and funnel plot are shown in Figures 3–5 and Supplementary Figures S3−S5. Then, we performed MR-PRESSO analysis to identify outlier SNPs and corrected the primary results. In the causal relationship between BW and BMI, rs1374204, rs2150052, rs12823128, and rs2229742 were identified as outliers, and the corrected results reached statistical significance (*p* = 3.19E-03). After removing rs17034876, rs1187118, rs11765649, rs1411424, rs10818797, rs2497304, rs72851023, rs7964361, and rs144843919, a negative relationship was observed between lower BW and SHBG (*p* = 1.24E-05). Outlier SNPS (rs11765649, rs12543725, rs72851023, and rs7964361) were deleted in the MR analysis of lower BW and bio-T, and the results were not altered (*p* = 1.67E-06). The rest of the sensitivity and MR-PRESSO analyses are shown in Supplementary Tables S3−S6. The causal effect of each SNP on the outcome is presented in Supplementary Tables S7−S17.

## Discussion

The present MR study clarified the genetic association between BW and female-related traits in the largest sample size of the European population. In this study, we found a positive effect of lower BW on bio-T, whereas LBW demonstrated an adverse effect on SHBG level. In contrast, we also found a causal effect of BW on BMI and lower BW on menarche but no detrimental effects of LBW on female-specific diseases. To the best of our knowledge, this is the first study to examine the likely causal relationship between BW and female traits and diseases based on hereditary information.

Our research results on infant BW and adult BMI are similar to those of most existing studies. Rogers (2003) and Zhao et al. (2012) provided good evidence of an association between high BW and subsequent BMI and an increased risk of overweight in young adults. In addition, Jelenkovic et al. (2017) and Liao et al. (2020) suggested a positive association between high BW and a later high BMI. For each individual, a 1.0 kg of BW increased, accompanied with a 0.33 or 0.9 kg/m^2^ of BMI increase in adulthood, respectively (*p* < 0.001). Moreover, factors such as genetics, development, and environment could result in individual variations in the concentrations of reproductive hormones. The association between lower BW and hyperandrogenism has been confirmed by almost all published evidence. Petraitiene et al. (2020) and Ruder et al. (2011) stated that small for gestational age or reduced BW girls demonstrated lower SHBG levels (*p* < 0.05) but higher concentrations of androstenedione, testosterone (T) (*p* < 0.05), dehydroepiandrosterone sulfate, and free androgen index (*p* < 0.01). Some studies showed that premature adrenarche results in an increased insulin response and hyperandrogenism in later adulthood (Szathmári et al., 2001; Schulte et al., 2016; Novello and Speiser, 2018). However, the relationship between lower BW and E_2_ levels remains controversial. Ruder et al. (2011) and Espetvedt Finstad et al. (2009) found an inverse association between BW and levels of E_2_, while Tworoger et al. (2006) and Sydsjö et al. (2019) demonstrated no differences in E_2_ levels between LBW women and controls. Although no direct evidence exists to prove the positive relationship between BW and E_2_, we might estimate an association between the ponderal index at birth/birth size and E_2_ (Jasienska et al., 2006; Espetvedt Finstad et al., 2009). Furthermore, our research was the same with that of Kerkhof et al. (2010) and Sydsjö et al. (2019), concluding that BW did not affect AMH concentrations. However, Dior et al. (2021) reported a significant association between lower BW and reduced AMH levels in 32-year-old women who were identified after adjusting for confounders (Î^2^ = 0.18, *p* < 0.05).

It is known that both genetic and environmental factors, such as smoking, body fat content, exposure to endocrine-disrupting chemicals, and BW, may result in early AAM (Behie and O'Donnell, 2015; Epplein et al., 2010; Żelaźniewicz et al., 2020; Adair, 2001). In our MR study, a surprising result was confirmed as well as in numerous observational studies. Moreover, Fan et al. (2018), Juul et al. (2017) , and Morris et al. (2010) discovered that lower BW in infancy may increase the risk of early menarche (*p* < 0.001). However, other studies found that no or an inverse relationship was found between BW and AAM (Sorensen et al., 2013; Sydsjö et al., 2019). Moreover, conclusions on LBW and ANM have not yet been unified, and positive (Alexander et al., 2014; Bjelland et al., 2020; Goldberg et al., 2020), or inverse (Tom et al., 2010), and even no relationship (A.Treloar et al., 2000) exist.

PCOS, endometriosis, and leiomyoma were regarded as the main female endocrine diseases that may affect reproduction. Although, we did not identify any causal effects of LBW on these three diseases. Few studies considered BW as an independent risk factor related to PCOS (Fulghesu et al., 2015), and they suggested that the high risk of PCOS and related traits are because of high BW (Cresswell et al., 1997; Michelmore et al., 2001). Almost all studies confirmed a correlation between LBW and endometriosis (Olšarová and Mishra, 2020). The studies of Gao et al. (2019), Gao et al. (2020) and Borghese et al. (Borghese et al., 2015) supported the fetal origins hypothesis of endometriosis (hazard ratio [HR] = 1.35, 95% CI = 1.08–1.67; OR = 1.5, 95% CI = 1.0–2.3, *p* < 0.05); even after adjusting for confounding factors, the results still remained (risk ratio = 1.3, 95% CI = 1.0–1.8, *p* < 0.05) (Missmer et al., 2004). Furthermore, the results of Aarestrup et al. (2020) and Wolff et al. (2013) studies did not reach statistical significance and confirmed this relationship. Last, limited evidence exists of an association between leiomyomas and BW (Wise et al., 2012).

The current study exhibited a few strengths. The major preponderance was the MR design, which cut down remaining confounders and reverse causality and, thereby, improved the causal inference in associations of lower BW with female-related traits. To avoid false-positive results and bias, we selected different sourced populations to eliminate overlapping, and two-sample MR analysis was performed *via* SNPs to analyze the causal relationship from exposure to outcomes. Next, the random allocation of individual genetic variation during gamete binding is used as an IV; thereby, MR analysis can largely avoid the influence of confounders, artificial errors, and bias and provide high-quality evidence. This MR analysis included sufficient samples and only included European participants to improve the dependability of the results. To conclude, the MVMR analysis was vital for exploring the direct correlation between LBW and female outcomes.

Next, inevitably, the present study demonstrates several limitations. First, GWAS were obtained only from European individuals in this study, whose results are not representative of other races or geographic areas. Second, parts of the summary dates were incomplete because of the privacy policy and long application period, which caused a lost partial population; in addition, the BW datasets obtained contained both males and females, which may lead to collider bias (Fry et al., 2017). To conclude, the present study mainly focused on the causal role of LBW on female-specific traits, but the underlying mechanisms remain to be elucidated.

## Conclusion

In summary, this analysis demonstrated that BW is positively associated with BMI in adulthood. In addition, LBW exhibits causal effects on decreased SHBG levels, increased bio-T levels, and early AAM.

## Data Availability

The original contributions presented in the study are included in the article/Supplementary Material, and further inquiries can be directed to the corresponding author.
