# Supplementary material for: The Causal Evidence of Birth Weight and Female-Related Traits and Diseases: A Two-Sample Mendelian Randomization Analysis
Source: Front Genet. 2022 Aug 12;13:850892. doi: 10.3389/fgene.2022.850892 (PMC9412024; doi:10.3389/fgene.2022.850892)
Supplement: Supplementary file 1 [file DataSheet1.docx]

| **Supplementary Table 1. Overview of exposure and 11 outcomes GWAS.** | | | |  |
| --- | --- | --- | --- | --- |
| **Traits** | **Consortium** | **(Case/Control) Sample Size** | **PMID** | **Female Only?** |
| **Exposure** |  |  |  |  |
| Birth Weight (BW) | Early Growth Genetics (EGG) | 143,677 | 31354785 | No |
|  |  |  | [27680694](https://www.ncbi.nlm.nih.gov/pubmed/27680694) |  |
| **Outcomes** |  |  |  |  |
| Body Mass Index (BMI) | UK biobank, GIANT | 379,501 | 30239722 | Yes |
| Total Testosterone (TT) | UK biobank | 230,454 | 32042192 | Yes |
| Bioavailable Testosterone (Bio-T) | UK biobank | 188,507 | 32042192 | Yes |
| Sex Hormone-Binding Globulin (SHBG) | UK biobank | 189,473 | 32042192 | Yes |
| Estradiol (E_2_) | UK biobank | 163,985 | 34255042 | Yes |
| Anti-Mullerian Hormone (AMH) | Genome-Wide Meta-Analysis | 3,344 | 30649302 | Yes |
| Menarche | MRC-IEU | 243,944 | ukb-b-3768 | Yes |
| Menopause | MRC-IEU | (153,820/57,294)  211,114 | ukb-b-18105 | Yes |
| Endometriosis | Finn Gen | (6,502/57,407)  63,909 | - | Yes |
| Polycystic Ovaries Syndrome (PCOS) | Genome-Wide Meta-Analysis | (4,138/20,129)  24,267 | 30566500 | Yes |
| Leiomyoma | Finn Gen | (14,569/72,789)  87,358 | - | Yes |

**Supplementary Material**

| **Supplementary Table 2. The description of studies used in the GWAS of birth weight** | | |
| --- | --- | --- |
| **Study** | **Sample Size (Male/Female)** | **Birth Weight (Mean±Sd, g)** |
| 1958 British Birth Cohort -UK | 4,595(2,320/2,275) | 3439±484 |
| ALSPACa, b - UK | 7,285(3,722/3,563) | 3553±491 |
| CHOP- Caucasian -USA | 9,405(5,040/4,365) | 3447±582 |
| CoLaus - Switzerland | 2,089(892/1,197) | 3490±668 |
| COPSAC-2000 -Denmark | 352(173/179) | NA |
| COPSAC-2010 -Denmark | 589(306/283) | 3635±483 |
| COPSAC-REGISTRY -Denmark | 1,210(804/406) | 3609±498 |
| DNBC -Denmark | 915(475/440) | 3767±480 |
| ERF -Netherlands | 459(187/272) | 3161±680 |
| EPIC -UK | 8,939(3,448/5,491) | 3505±786 |
| Fenland (GA+) -UK | 5,188(2,088/3,100) | 3433±638 |
| Fenland (GA-) -UK | 833(509/324) | 3465±593 |
| Generation R -Netherlands | 2,701(1,378/1,323) | 3628±494 |
| GINIplus & LISAplus (GA+) -Germany | 656(360/296) | 3498±406 |
| GINIplus & LISAplus (GA-) -Germany | 790(391/399) | 3499±429 |
| GOYA -Denmark | 149/0 (obese), 141/0 (control) | NA |
| HBCS -Finland | 1472(639/833) | 3536±460 |
| INMA -Spain | 1,021(527/494) | 3362±406 |
| INTER99 -Denmark | 4,243(1,981/2,262) | 3505±493 |
| Leipzig -Germany | 597 (304/293) | 3573±531 |
| NEO -Netherlands | 504 (exact; 200/304), 3215 (range; 1,450/1,765) | 3669±1068 |
| NFBC1966 -Finland | 5,009(2,393/2,616) | 3607±506 |
| NFBC1986 -Finland | 4,680(2,306/2,374) | 3626±543 |
| NTR -Netherlands | 1,265(447/818) | 3414±619 |
| ORCADES -Scotland | 960(330/630) | 3401±607 |
| PANIC -Finland | 436(231/205) | 3646±488 |
| RAINE -Australia | 1,347(693/654) | 3505±471 |
| SORBS -Germany | 298(113/185) | N/A |
| STRIP -Finland | 599(311/288) | 3696±471 |
| TEENAGE (GA+) -Greece | 279(126/153) | 3403±467 |
| TEENAGE (GA-) -Greece | 551(234/317) | 3398±459 |
| TDCOB-cases -Denmark | 669(391/278) | 3682±536 |
| TDCOB-controls -Denmark | 560(211/349) | 3627±517 |
| YFS -Finland | 1,915(861/1,054) | 3648±491 |
| UK BioBank -UK | 67,786(40,425/27,361) | 3452±416 |
| Total | 143,702 (76,556/67,146) | 3,449±488 |

All GWAS data were collected from the study of Horikoshi et al., 2016 and participants were all European individuals, the table described the country, sex, and birthweight (mean and standard deviation (SD) in grams) of 35 studies, which included total 143,702 samples (male 76,556 and female 67,146). And the mean±SD for birth weight were 3,449±488g , respectively.

NA: Not Applicable

| **Supplementary Table 2. The characteristics of 48 BW associated SNPs** | | | | | | | | |
| --- | --- | --- | --- | --- | --- | --- | --- | --- |
| **SNP** | **Chromosome: Position** | **Nearest Gene** | **EA (OA)** | **EAF** | **BW-BETA** | **LBW-BETA** | **SE** | **P-value** |
| rs138715366 | 7:44246271 | YKT6-GCK | T(C) | 0.01 | -0.2412 | 0.2412 | 0.0229 | 7.20E-26 |
| rs144843919 | 17:29037339 | SUZ12P1-CRLF3 | A(G) | 0.04 | -0.066 | 0.066 | 0.0116 | 1.4E-08 |
| rs13322435 | 3:156795468 | CCNL1-LEKR1 | G(A) | 0.4 | -0.0533 | 0.0533 | 0.004 | 3.60E-41 |
| rs35261542 | 6:20675792 | CDKAL1 | A(C) | 0.27 | -0.0444 | 0.0444 | 0.0041 | 4.40E-27 |
| rs1351394 | 12:66351826 | HMGA2 | C(T) | 0.51 | -0.0436 | 0.0436 | 0.0037 | 1.90E-32 |
| rs7575873 | 2:23962647 | ATAD2B | G(A) | 0.12 | -0.0384 | 0.0384 | 0.0057 | 1.20E-11 |
| rs1101081 | 6:152032917 | ESR1 | T(C) | 0.28 | -0.0376 | 0.0376 | 0.0042 | 1.60E-19 |
| rs2229742 | 21:16339172 | NRIP1 | C(G) | 0.13 | -0.036 | 0.036 | 0.006 | 2.20E-09 |
| rs925098 | 4:17919811 | LCORL | A(G) | 0.73 | -0.034 | 0.034 | 0.0042 | 5.40E-16 |
| rs700059 | 9:125824055 | STRBP | A(G) | 0.86 | -0.0334 | 0.0334 | 0.0054 | 4.70E-10 |
| rs13266210 | 8:41533514 | ANK1-NKX6-3 | G(A) | 0.21 | -0.0308 | 0.0308 | 0.0045 | 1.30E-11 |
| rs61862780 | 10:94468643 | HHEX-IDE | C(T) | 0.49 | -0.0281 | 0.0281 | 0.0037 | 3.00E-14 |
| rs854037 | 5:57091783 | 5q11.2 | G(A) | 0.19 | -0.0268 | 0.0268 | 0.0048 | 2.2E-08 |
| rs11765649 | 7:23479013 | IGF2BP3 | C(T) | 0.25 | -0.0267 | 0.0267 | 0.0043 | 5.80E-10 |
| rs28530618 | 20:31275581 | C20orl203 | G(A) | 0.51 | -0.0261 | 0.0261 | 0.0038 | 7.70E-12 |
| rs1415701 | 6:130345835 | L3MBTL3 | A(G) | 0.26 | -0.0253 | 0.0253 | 0.0043 | 2.60E-09 |
| rs139975827 | 12:22068161 | ABCC9 | A(G) | 0.38 | -0.0248 | 0.0248 | 0.0043 | 1.1E-08 |
| rs798489 | 7:2801803 | GNA12 | T(C) | 0.27 | -0.0233 | 0.0233 | 0.0042 | 2.00E-08 |
| rs7402982 | 15:99193269 | IGF1R | G(A) | 0.57 | -0.0232 | 0.0232 | 0.0039 | 2.30E-09 |
| rs12543725 | 8:142247979 | SLC45A4 | A(G) | 0.41 | -0.0231 | 0.0231 | 0.0038 | 1.20E-09 |
| rs6959887 | 7:35295365 | TBX20 | G(A) | 0.39 | -0.0228 | 0.0228 | 0.0038 | 1.50E-09 |
| rs12906125 | 15:91427612 | FES | A(G) | 0.32 | -0.0228 | 0.0228 | 0.004 | 1.7E-08 |
| rs134594 | 22:29468456 | KREMEN1 | T(C) | 0.65 | -0.0227 | 0.0227 | 0.004 | 1.00E-08 |
| rs10935733 | 3:148622968 | CPA3 | C(T) | 0.59 | -0.0221 | 0.0221 | 0.0039 | 9.20E-09 |
| rs2242116 | 3:46941116 | PTH1R | G(A) | 0.62 | -0.0216 | 0.0216 | 0.0038 | 1.4E-08 |
| rs12823128 | 12:26872730 | ITPR2 | C(T) | 0.46 | -0.0211 | 0.0211 | 0.0037 | 1.9E-08 |
| rs2421016 | 10:124167512 | PLEKHA1 | T(C) | 0.49 | 0.0207 | -0.0207 | 0.0037 | 1.8E-08 |
| rs2150052 | 9:113945067 | LPAR1 | T(A) | 0.5 | 0.0211 | -0.0211 | 0.0038 | 2.2E-08 |
| rs6040076 | 20:10658882 | JAG1 | C(G) | 0.49 | 0.0231 | -0.0231 | 0.0039 | 2E-09 |
| rs10830963 | 11:92708710 | MTNR1B | G(C) | 0.28 | 0.0232 | -0.0232 | 0.0042 | 2.9E-08 |
| rs7847628 | 9:123631225 | PHF19 | G(A) | 0.68 | 0.0233 | -0.0233 | 0.0041 | 1.00E-08 |
| rs6016377 | 20:39172728 | MAFB | T(C) | 0.43 | 0.0239 | -0.0239 | 0.0039 | 9.50E-10 |
| rs7729301 | 5:157886953 | EBF1 | A(G) | 0.73 | 0.0239 | -0.0239 | 0.0042 | 1.6E-08 |
| rs6537307 | 4:145601863 | HHIP | G(A) | 0.5 | 0.0254 | -0.0254 | 0.0037 | 9.50E-12 |
| rs7742369 | 6:34165721 | HMGA1 | G(A) | 0.18 | 0.0283 | -0.0283 | 0.0049 | 9.90E-09 |
| rs3753639 | 1:154986091 | ZBTB7B | C(T) | 0.24 | 0.0306 | -0.0306 | 0.0045 | 7.30E-12 |
| rs113086489 | 17:7171356 | CLDN7 | T(C) | 0.56 | 0.0307 | -0.0307 | 0.0038 | 9.10E-16 |
| rs72480273 | 1:161644871 | FCGR2B | C(A) | 0.17 | 0.0313 | -0.0313 | 0.0051 | 8.00E-10 |
| rs2473248 | 1:22536643 | WNT4-ZBTB40 | C(T) | 0.87 | 0.0325 | -0.0325 | 0.0057 | 1.00E-09 |
| rs1819436 | 13:78580283 | RNF219-AS1 | C(T) | 0.87 | 0.0329 | -0.0329 | 0.0057 | 6.30E-09 |
| rs7076938 | 10:115789375 | ADRB1 | T(C) | 0.73 | 0.0363 | -0.0363 | 0.0042 | 4.70E-18 |
| rs7964361 | 12:102994878 | IGF1 | A(G) | 0.09 | 0.0391 | -0.0391 | 0.0067 | 4.70E-09 |
| rs11719201 | 3:123068744 | ADCY5 | T(C) | 0.23 | 0.0463 | -0.0463 | 0.0044 | 2.40E-26 |
| rs1374204 | 2:46484205 | EPAS1 | T(C) | 0.7 | 0.047 | -0.047 | 0.0042 | 6.20E-29 |
| rs62240962 | 22:42259524 | SREBF2 | C(T) | 0.08 | 0.047 | -0.047 | 0.007 | 9.70E-12 |
| rs72851023 | 11:2130620 | INS-IGF2 | T(C) | 0.07 | 0.0476 | -0.0476 | 0.0075 | 2.90E-10 |
| rs62466330 | 7:73056805 | MLXIPL | C(T) | 0.07 | 0.0486 | -0.0486 | 0.0075 | 1.20E-10 |
| rs28510415 | 9:98245026 | PTCH1 | G(A) | 0.09 | 0.0557 | -0.0557 | 0.0065 | 1.50E-17 |

| **Supplementary Table 3. Comparison of the different statistical methods for MR analysis evaluating the causal association between BW/lower BW and female-related traits.** | | | | | | | |
| --- | --- | --- | --- | --- | --- | --- | --- |
| **Exposure-Outcome** | **Method** | **LBW (47 SNP)** | | | **LBW (48 SNP)** | | |
|  |  | **β[OR]** | **SE [95%CI]** | **P-value** | **β[OR]** | **SE [95%CI]** | **P-value** |
| BW-BMI | MR Egger | 0.018 | 0.080 | 8.19E-01 | -0.017 | 0.075 | 8.19E-01 |
|  | Weighted median | 0.043 | 0.023 | 6.75E-02 | 0.044 | 0.023 | 5.71E-02 |
|  | IVW | 0.056 | 0.028 | 4.25E-02 | 0.071 | 0.027 | **7.63E-03** |
|  | Weighted mode | 0.018 | 0.029 | 5.50E-01 | 0.030 | 0.028 | 2.83E-01 |
|  | MR-PRESSO | 0.034 | 0.021 | 8.50E-02 | 0.071 | 0.075 | **3.19E-03** |
| LBW-E_2_ | MR Egger | 0.018 | 0.256 | 9.44E-01 | -0.049 | 0.242 | 8.41E-01 |
|  | Weighted median | 0.019 | 0.107 | 8.62E-01 | -0.013 | 0.101 | 8.98E-01 |
|  | IVW | -0.030 | 0.075 | 6.89E-01 | -0.052 | 0.072 | 4.70E-01 |
|  | Weighted mode | 0.048 | 0.187 | 8.01E-01 | -0.049 | 0.242 | 8.41E-01 |
| LBW-SHBG | MR Egger | -0.025 | 0.048 | 5.95E-01 | -0.060 | 0.054 | 2.75E-01 |
|  | Weighted median | -0.051 | 0.014 | 4.38E-04 | -0.051 | 0.016 | 1.04E-03 |
|  | IVW | -0.081 | 0.017 | **2.08E-06** | -0.075 | 0.019 | **9.36E-05** |
|  | Weighted mode | -0.042 | 0.022 | 5.82E-02 | -0.048 | 0.023 | 4.28E-02 |
|  | MR-PRESSO | -0.067 | 0.013 | **1.24E-05** | -0.063 | 0.013 | **3.03E-05** |
| LBW-Bio-T | MR Egger | 0.080 | 0.068 | 2.51E-01 | 0.135 | 0.077 | 8.70E-02 |
|  | Weighted median | 0.091 | 0.024 | 1.67E-04 | 0.092 | 0.024 | 1.28E-04 |
|  | IVW | 0.105 | 0.024 | **1.25E-05** | 0.103 | 0.027 | **1.42E-04** |
|  | Weighted mode | 0.083 | 0.052 | 1.17E-01 | 0.108 | 0.046 | 2.22E-02 |
|  | MR-PRESSO | 0.103 | 0.018 | **1.67E-06** | 0.103 | 0.019 | **3.17E-06** |

| **Continued Supplementary Table 3** | | | | | | | |
| --- | --- | --- | --- | --- | --- | --- | --- |
| **Exposure-Outcome** | **Method** | **LBW (47 SNP)** | | | **LBW (48 SNP)** | | |
|  |  | **β[OR]** | **SE [95%CI]** | **P-value** | **β[OR]** | **SE [95%CI]** | **P-value** |
| LBW-TT | MR Egger | 0.072 | 0.115 | 5.33E-01 | 0.077 | 0.113 | 4.96E-01 |
|  | Weighted median | 0.023 | 0.029 | 4.35E-01 | 0.020 | 0.030 | 4.96E-01 |
|  | IVW | 0.031 | 0.041 | 4.44E-01 | 0.033 | 0.040 | 4.11E-01 |
|  | Weighted mode | 0.017 | 0.065 | 7.89E-01 | 0.014 | 0.064 | 8.34E-01 |
|  | MR-PRESSO | - | - | - | -0.018 | 0.025 | 4.59E-01 |
| LBW-AMH | MR Egger | 0.430 | 0.323 | 1.91E-01 | 0.321 | 0.316 | 3.16E-01 |
|  | Weighted median | 0.001 | 0.160 | 9.95E-01 | -0.024 | 0.159 | 8.79E-01 |
|  | IVW | 0.063 | 0.114 | 5.85E-01 | 0.039 | 0.113 | 7.30E-01 |
|  | Weighted mode | 0.039 | 0.243 | 8.73E-01 | -0.039 | 0.232 | 8.66E-01 |
| LBW-Menarche | MR Egger | -0.024 | 0.061 | 6.94E-01 | -0.057 | 0.058 | 3.36E-01 |
|  | Weighted median | -0.018 | 0.020 | 3.57E-01 | -0.018 | 0.020 | 3.63E-01 |
|  | IVW | -0.050 | 0.021 | 1.91E-02 | -0.053 | 0.021 | **9.82E-03** |
|  | Weighted mode | 0.005 | 0.038 | 8.96E-01 | 0.005 | 0.041 | 8.99E-01 |
|  | MR-PRESSO | -0.048 | 0.016 | **4.75E-03** | -0.034 | 0.015 | 3.33E-02 |
| LBW-Menopause | MR Egger | -0.020 | 0.025 | 4.29E-01 | -0.035 | 0.025 | 1.58E-01 |
|  | Weighted median | 0.007 | 0.011 | 5.57E-01 | 0.000 | 0.012 | 9.70E-01 |
|  | IVW | 0.010 | 0.009 | 2.70E-01 | 0.010 | 0.009 | 2.73E-01 |
|  | Weighted mode | -0.021 | 0.025 | 3.93E-01 | -0.019 | 0.024 | 4.32E-01 |
|  | MR-PRESSO | - | - | - | 0.013 | 0.008 | 1.09E-01 |

| **Continued Supplementary Table 3** | | | | |  |  |  |
| --- | --- | --- | --- | --- | --- | --- | --- |
| **Exposure-Outcome** | **Method** | **LBW (47 SNP)** | | | **LBW (48 SNP)** | | |
|  |  | **β[OR]** | **SE [95%CI]** | **P-value** | **β[OR]** | **SE [95%CI]** | **P-value** |
| LBW-Endometriosis | MR Egger | 2.998 | [1.028-8.743] | 5.07E-02 | 1.125 | [1.140-8.319] | 3.21E-02 |
|  | Weighted median | 0.987 | [0.708-1.376] | 9.38E-01 | 0.060 | [0.759-1.484] | 7.27E-01 |
|  | IVW | 1.107 | [0.785-1.562] | 5.63E-01 | 0.136 | [0.828-1.586] | 4.12E-01 |
|  | Weighted mode | 1.046 | [0.562-1.947] | 8.88E-01 | 0.082 | [0.602-1.957] | 7.87E-01 |
|  | MR-PRESSO | 1.021 | [0.980-1.065] | 8.84E-01 | 1.059 | [0.824-1.362] | 6.55E-01 |
| LBW-Leiomyoma | MR Egger | 0.801 | [0.403-1.592] | 5.30E-01 | 0.974 | [0.465-2.041] | 9.45E-01 |
|  | Weighted median | 0.924 | [0.710-1.202] | 5.55E-01 | 0.891 | [0.684-1.161] | 3.94E-01 |
|  | IVW | 0.842 | [0.682-1.039] | 1.09E-01 | 0.791 | [0.629-0.994] | 4.46E-02 |
|  | Weighted mode | 0.908 | [0.584-1.411] | 6.71E-01 | 0.882 | [0.579-1.345] | 5.64E-01 |
|  | MR-PRESSO | 0.866 | [0.708-1.060] | 1.69 E-01 | - | - | - |
| LBW-PCOS | MR Egger | 0.765 | [0.171-3.431] | 7.29E-01 | 1.178 | [0.291-4.763] | 8.19E-01 |
|  | Weighted median | 1.163 | [0.670-2.017] | 5.91E-01 | 0.945 | [0.556-1.609] | 8.36E-01 |
|  | IVW | 1.362 | [0.879-2.110] | 1.67E-01 | 1.125 | [0.748-1.691] | 5.72E-01 |
|  | Weighted mode | 1.183 | [0.520-2.691] | 6.90E-01 | 1.031 | [0.478-2.226] | 9.38E-01 |
|  | MR-PRESSO | 1.511 | [1.007-2.267] | 6.90E-01 | - | - | - |

IVW: Inverse variance weighted; BMI: Body Mass Index; E2: Estradiol; SHBG: Sex Hormone-Binding Globulin; TT: Total Testosterone; Bio-TT: Bio-Available Testosterone; AMH: Anti-Mullerian Hormone; PCOS: Polycystic Ovaries Syndrome. Red p-value：p<0.05; Red and bold p-value: p<4.55E-3.p-valu; LBW (47 SNP): Exposure-SNPs of plink version from Ping Zeng et al; LBW (48 SNP): Exposure-SNPs of primary version from Horikoshi M et al.

| **Supplementary Table 4. Heterogeneity estimation for each group** | | | | | | | | |
| --- | --- | --- | --- | --- | --- | --- | --- | --- |
| **Outcome** | **Method** | **BW/LBW (47 SNP)** | | | | **BW/LBW (48 SNP)** | | |
|  |  | **Q** | **Q_df** | **Q_pval** | **Q** | | **Q_df** | **Q_pval** |
| BMI | MR-Egger | 159.878 | 39 | 1.42E-16 | 143.774 | | 38 | 3.28E-14 |
|  | IVW | 160.926 | 40 | 1.96E-16 | 149.754 | | 39 | 6.84E-15 |
| E_2_ | MR-Egger | 25.452 | 34 | 8.55E-01 | 25.289 | | 35 | 8.86E-01 |
|  | IVW | 25.491 | 35 | 8.81E-01 | 25.289 | | 36 | 9.09E-01 |
| SHBG | MR-Egger | 202.647 | 43 | 1.46E-22 | 286.791 | | 44 | 2.35E-37 |
|  | IVW | 209.928 | 44 | 1.75E-23 | 287.342 | | 45 | 4.79E-37 |
| Bio-T | MR-Egger | 130.728 | 43 | 8.75E-11 | 180.294 | | 44 | 2.04E-18 |
|  | IVW | 131.213 | 44 | 1.31E-10 | 181.075 | | 45 | 3.09E-18 |
| TT | MR-Egger | 303.082 | 43 | 8.04E-41 | 316.405 | | 44 | 6.76E-43 |
|  | IVW | 304.124 | 44 | 1.38E-40 | 317.704 | | 45 | 1.04E-42 |
| AMH | MR-Egger | 26.5231 | 39 | 9.36E-01 | 28.952 | | 38 | 8.54E-01 |
|  | IVW | 28.003 | 40 | 9.23E-01 | 29.864 | | 39 | 8.53E-01 |
| Menarche | MR-Egger | 156.016 | 43 | 9.90E-15 | 133.776 | | 40 | 4.89E-12 |
|  | IVW | 156.775 | 44 | 1.45E-14 | 133.791 | | 41 | 9.04E-12 |
| Menopause | MR-Egger | 59.597 | 43 | 4.74E-02 | 54.545 | | 40 | 6.24E-02 |
|  | IVW | 61.852 | 44 | 3.90E-02 | 59.866 | | 41 | 2.87E-02 |
| Endometriosis | MR-Egger | 102.418 | 43 | 9.35E-07 | 87.464 | | 41 | 3.27E-05 |
|  | IVW | 111.197 | 44 | 9.79E-08 | 96.470 | | 42 | 3.60E-06 |
| Leiomyoma | MR-Egger | 74.509 | 43 | 2.03E-03 | 78.672 | | 40 | 2.52E-04 |
|  | IVW | 74.548 | 44 | 2.73E-03 | 79.335 | | 41 | 3.06E-04 |
| PCOS | MR-Egger | 68.117 | 43 | 8.68E-03 | 62.425 | | 43 | 2.79E-02 |
|  | IVW | 69.099 | 44 | 9.21E-03 | 62.431 | | 44 | 3.50E-02 |

IVW: Inverse variance weighted; BMI: Body Mass Index; E2: Estradiol; SHBG: Sex Hormone-Binding Globulin; TT: Total Testosterone; Bio-TT: Bio-Available Testosterone; AMH: Anti-Mullerian Hormone; PCOS: Polycystic Ovaries Syndrome.

| **Supplementary Table 5. Summary for directional horizontal pleiotropy tests** | | | | | | | | |
| --- | --- | --- | --- | --- | --- | --- | --- | --- |
| **Outcome** | **Method** | **BW/LBW (47 SNP)** | | | **BW/LBW (48 SNP)** | | | |
|  |  | **Effect** | **SE** | **P-value** | | **Effect** | **SE** | **P-value** |
| BMI | MR-Egger Intercept | 0.001 | 0.003 | 6.16 E-01 | | 0.003 | 0.002 | 2.16E-01 |
| E_2_ | MR-Egger Intercept | -0.002 | 0.008 | 8.45E-01 | | 0.000 | 0.008 | 9.89E-01 |
| SHBG | MR-Egger Intercept | -0.002 | 0.002 | 2.21E-01 | | -0.001 | 0.002 | 7.73E-01 |
| Bio-T | MR-Egger Intercept | 0.001 | 0.002 | 6.92E-01 | | -0.001 | 0.003 | 6.65E-01 |
| TT | MR-Egger Intercept | -0.001 | 0.004 | 7.02E-01 | | -0.002 | 0.004 | 6.73E-01 |
| AMH | MR-Egger Intercept | -0.013 | 0.011 | 2.31E-01 | | -0.010 | 0.011 | 3.46E-01 |
| Menarche | MR-Egger Intercept | -0.001 | 0.002 | 6.50E-01 | | 0.000 | 0.002 | 9.48E-01 |
| Menopause | MR-Egger Intercept | 0.001 | 0.001 | 2.09E-01 | | 0.002 | 0.001 | 5.51E-02 |
| Endometriosis | MR-Egger Intercept | -0.033 | 0.017 | 6.15E-02 | | -0.034 | 0.016 | 4.63E-02 |
| Leiomyoma | MR-Egger Intercept | 0.002 | 0.011 | 8.82E-01 | | -0.007 | 0.012 | 5.65E-01 |
| PCOS | MR-Egger Intercept | 0.019 | 0.024 | 4.35E-01 | | -0.002 | 0.023 | 9.46E-01 |

| **Supplementary Table 6.** Multivariable MR analysis to adjust the causal effect of BMI on BW | | | | | | |
| --- | --- | --- | --- | --- | --- | --- |
| **Exposure** | **Traits** | **Method** | **N SNPs** | **β[OR]** | **SE [95%CI]** | **P-value** |
| LBW  BMI | SHBG | IVW | 18 | -0.112 | [-0.172--0.051] | 2.80E-04 |
|  |  |  | 456 | -0.181 | [-0.202--0.160] | 4.32E-64 |
|  | Bio-T | IVW | 18 | 0.145 | [0.064-0.225] | 4.21E-04 |
|  |  |  | 456 | 0.252 | [0.223-0.281] | 4.00E-66 |
|  | Menarche | IVW | 18 | -0.096 | [-0.179--0.013] | 2.26E-02 |
|  |  |  | 456 | -0.271 | [-0.301--0.241] | 1.20E-69 |
|  | Leiomyoma | IVW | 18 | 1.030 | [0.766-1.385] | 8.44E-01 |
|  |  |  | 440 | 1.051 | [0.943-1.170] | 3.70E-01 |

IVW and MR-Egger regression are two-sided statistical tests.

IVW inverse variance weighted, OR odds ratio, 95%CI: 95% confidence intervals.

*The previous row means the effect of LBW on each trait while next line meaning the effect of BMI on traits in our MVMR analysis.

| **Supplementary Table 7. The genetic associations between the BW on female BMI.** | | | | | | | | | | | | | |
| --- | --- | --- | --- | --- | --- | --- | --- | --- | --- | --- | --- | --- | --- |
| **SNP (47)** | **EA** | **OA** | **EAF** | **β** | **SE** | **P-value** | **SNP (48)** | **EA** | **OA** | **EAF** | **β** | **SE** | **P-value** |
| rs2473248 | T | C | 0.122 | -0.004 | 0.004 | 3.56E-01 | rs2473248 | T | C | 0.122 | -0.004 | 0.004 | 3.56E-01 |
| rs3753639 | T | C | 0.760 | -0.017 | 0.003 | 6.63E-08 | rs3753639 | T | C | 0.760 | -0.017 | 0.003 | 6.63E-08 |
| rs72480273 | A | C | 0.816 | -0.003 | 0.003 | 4.58E-01 | rs72480273 | A | C | 0.816 | -0.003 | 0.003 | 4.58E-01 |
| rs17034876 | T | C | 0.674 | 0.003 | 0.003 | 2.04E-01 | rs1374204 | T | C | 0.674 | 0.003 | 0.003 | 2.19E-01 |
| rs2168443 | A | T | 0.621 | 0.004 | 0.003 | 9.97E-02 | rs2242116 | A | G | 0.378 | -0.003 | 0.002 | 2.45E-01 |
| rs11719201 | T | C | 0.242 | 0.002 | 0.003 | 5.12E-01 | rs11719201 | T | C | 0.242 | 0.002 | 0.003 | 5.12E-01 |
| rs10935733 | T | C | 0.401 | 0.001 | 0.003 | 7.93E-01 | rs10935733 | T | C | 0.401 | 0.001 | 0.003 | 7.93E-01 |
| rs900399 | A | G | 0.606 | -0.002 | 0.002 | 4.47E-01 | rs13322435 | A | G | 0.590 | -0.001 | 0.002 | 6.60E-01 |
| rs4144829 | T | C | 0.738 | 0.007 | 0.003 | 2.34E-02 | rs925098 | A | G | 0.714 | 0.006 | 0.003 | 1.44E-02 |
| rs2131354 | A | G | 0.517 | 0.003 | 0.003 | 2.13E-01 | rs6537307 | A | G | 0.521 | -0.006 | 0.002 | 1.10E-02 |
| rs854037 | A | G | 0.806 | 0.000 | 0.003 | 8.82E-01 | rs854037 | A | G | 0.806 | 0.000 | 0.003 | 8.82E-01 |
| rs2946179 | T | C | 0.268 | -0.009 | 0.003 | 4.15E-03 | rs7729301 | A | G | 0.732 | 0.008 | 0.003 | 4.37E-03 |
| rs35261542 | A | C | 0.262 | -0.005 | 0.003 | 9.07E-02 | rs35261542 | A | C | 0.262 | -0.005 | 0.003 | 9.07E-02 |
| rs9368777 | C | G | 0.576 | -0.004 | 0.003 | 1.02E-01 | rs7742369 | A | G | 0.830 | -0.007 | 0.003 | 1.37E-02 |
| rs1187118 | A | T | 0.173 | 0.006 | 0.003 | 6.15E-02 | rs1415701 | A | G | 0.265 | -0.009 | 0.003 | 3.40E-04 |
| rs10872678 | T | C | 0.743 | -0.004 | 0.002 | 9.09E-02 | rs1101081 | T | C | 0.270 | 0.004 | 0.003 | 1.53E-01 |
| rs798498 | T | G | 0.701 | 0.000 | 0.003 | 9.61E-01 | rs798489 | T | C | 0.265 | -0.002 | 0.003 | 4.38E-01 |
| rs138715366 | T | C | 0.008 | -0.011 | 0.015 | 4.53E-01 | rs6959887 | A | G | 0.629 | -0.005 | 0.002 | 4.15E-02 |
| rs111778406 | A | G | 0.930 | 0.004 | 0.005 | 4.25E-01 | rs138715366 | T | C | 0.008 | -0.011 | 0.015 | 4.53E-01 |
| rs13266210 | A | G | 0.800 | 0.002 | 0.003 | 5.89E-01 | rs62466330 | T | C | 0.933 | 0.004 | 0.005 | 4.95E-01 |
| rs12543725 | A | G | 0.407 | 0.001 | 0.002 | 7.40E-01 | rs13266210 | A | G | 0.800 | 0.002 | 0.003 | 5.89E-01 |
| rs7854962 | C | G | 0.787 | -0.006 | 0.003 | 5.25E-02 | rs12543725 | A | G | 0.407 | 0.001 | 0.002 | 7.40E-01 |
| rs3780573 | A | G | 0.095 | -0.002 | 0.005 | 6.33E-01 | rs28510415 | A | G | 0.909 | 0.004 | 0.005 | 3.81E-01 |
| rs1411424 | A | G | 0.523 | 0.000 | 0.002 | 8.69E-01 | rs2150052 | A | T | 0.490 | -0.001 | 0.002 | 5.46E-01 |
| rs10818797 | T | C | 0.845 | 0.011 | 0.003 | 7.00E-04 | rs700059 | A | G | 0.847 | 0.011 | 0.003 | 4.91E-04 |
| rs2497304 | T | C | 0.470 | -0.007 | 0.002 | 2.44E-03 | rs61862780 | T | C | 0.522 | 0.008 | 0.003 | 3.42E-03 |
| rs79237883 | T | C | 0.920 | -0.014 | 0.005 | 4.50E-03 | rs7076938 | T | C | 0.711 | -0.001 | 0.003 | 7.46E-01 |
| rs740746 | A | G | 0.711 | 0.000 | 0.003 | 9.71E-01 | rs2421016 | T | C | 0.457 | 0.005 | 0.002 | 3.02E-02 |
| rs72851023 | T | C | 0.074 | 0.016 | 0.005 | 1.41E-03 | rs72851023 | T | C | 0.074 | 0.016 | 0.005 | 1.41E-03 |
| rs2306547 | T | C | 0.478 | -0.001 | 0.002 | 6.35E-01 | rs10830963 | C | G | 0.720 | -0.009 | 0.003 | 3.40E-04 |
| rs7964361 | A | G | 0.085 | -0.003 | 0.004 | 4.25E-01 | rs12823128 | T | C | 0.522 | 0.001 | 0.002 | 6.39E-01 |
| rs7998537 | A | G | 0.303 | 0.003 | 0.003 | 2.10E-01 | rs1351394 | T | C | 0.481 | 0.002 | 0.002 | 3.85E-01 |
| rs1819436 | T | C | 0.125 | -0.005 | 0.004 | 1.79E-01 | rs7964361 | A | G | 0.085 | -0.003 | 0.004 | 4.25E-01 |
| rs28415607 | T | C | 0.756 | -0.016 | 0.003 | 4.71E-07 | rs1819436 | T | C | 0.125 | -0.005 | 0.004 | 1.79E-01 |
| rs113086489 | T | C | 0.541 | 0.011 | 0.003 | 3.35E-05 | rs113086489 | T | C | 0.541 | 0.011 | 0.003 | 3.35E-05 |
| rs144843919 | A | G | 0.036 | -0.004 | 0.007 | 5.39E-01 | rs144843919 | A | G | 0.036 | -0.004 | 0.007 | 5.39E-01 |
| rs72833480 | A | G | 0.290 | -0.003 | 0.003 | 2.52E-01 | rs6040076 | C | G | 0.510 | 0.000 | 0.003 | 8.93E-01 |
| rs6040076 | C | G | 0.510 | 0.000 | 0.003 | 8.93E-01 | rs28530618 | A | G | 0.480 | -0.001 | 0.003 | 6.26E-01 |
| rs28530618 | A | G | 0.480 | -0.001 | 0.003 | 6.26E-01 | rs6016377 | T | C | 0.446 | 0.006 | 0.002 | 1.20E-02 |
| rs6016377 | T | C | 0.446 | 0.006 | 0.002 | 1.20E-02 | rs2229742 | C | G | 0.100 | -0.005 | 0.004 | 1.48E-01 |
| rs2229742 | C | G | 0.100 | -0.005 | 0.004 | 1.48E-01 | rs134594 | T | C | 0.666 | -0.002 | 0.002 | 3.63E-01 |
| rs134594 | T | C | 0.666 | -0.002 | 0.002 | 3.63E-01 | rs62240962 | T | C | 0.079 | -0.017 | 0.005 | 4.93E-04 |
| rs41311445 | A | C | 0.908 | 0.017 | 0.005 | 1.99E-04 |  |  |  |  |  |  |  |

| **Supplementary Table 8. The genetic associations between the lower BW on E_2_.** | | | | | | | | | | | | | | |
| --- | --- | --- | --- | --- | --- | --- | --- | --- | --- | --- | --- | --- | --- | --- |
| **SNP (47)** | **EA** | **OA** | **EAF** | **β** | **SE** | **P-value** | **SNP (48)** | **EA** | **OA** | **EAF** | **β** | **SE** | **P-value** |  |
| rs2473248 | T | C | 0.118 | -0.017 | 0.019 | 3.77E-01 | rs2473248 | T | C | 0.118 | -0.017 | 0.019 | 3.77E-01 |  |
| rs3753639 | C | T | 0.245 | -0.012 | 0.014 | 3.90E-01 | rs3753639 | C | T | 0.245 | -0.012 | 0.014 | 3.90E-01 |  |
| rs72480273 | C | A | 0.191 | 0.015 | 0.016 | 3.28E-01 | rs72480273 | C | A | 0.191 | 0.015 | 0.016 | 3.28E-01 |  |
| rs79237883 | C | T | 0.076 | 0.026 | 0.023 | 2.51E-01 | rs61862780 | C | T | 0.492 | 0.004 | 0.012 | 7.57E-01 |  |
| rs740746 | G | A | 0.266 | -0.029 | 0.014 | 3.60E-02 | rs7076938 | C | T | 0.266 | -0.029 | 0.014 | 3.70E-02 |  |
| rs72851023 | T | C | 0.077 | 0.010 | 0.023 | 6.75E-01 | rs2421016 | T | C | 0.470 | 0.000 | 0.012 | 9.80E-01 |  |
| rs7964361 | A | G | 0.089 | -0.043 | 0.021 | 4.10E-02 | rs72851023 | T | C | 0.077 | 0.010 | 0.023 | 6.75E-01 |  |
| rs7998537 | A | G | 0.320 | -0.006 | 0.013 | 6.60E-01 | rs10830963 | G | C | 0.274 | -0.009 | 0.013 | 4.93E-01 |  |
| rs1819436 | T | C | 0.123 | 0.005 | 0.018 | 7.88E-01 | rs1351394 | T | C | 0.491 | 0.016 | 0.012 | 1.96E-01 |  |
| rs12906125 | A | G | 0.327 | 0.000 | 0.013 | 9.99E-01 | rs7964361 | A | G | 0.089 | -0.043 | 0.021 | 4.06E-02 |  |
| rs28415607 | C | T | 0.247 | -0.007 | 0.014 | 6.39E-01 | rs1819436 | T | C | 0.123 | 0.005 | 0.018 | 7.88E-01 |  |
| rs113086489 | C | T | 0.454 | -0.005 | 0.012 | 6.61E-01 | rs12906125 | A | G | 0.327 | 0.000 | 0.013 | 9.99E-01 |  |
| rs144843919 | A | G | 0.036 | -0.013 | 0.033 | 6.98E-01 | rs113086489 | C | T | 0.454 | -0.005 | 0.012 | 6.61E-01 |  |
| rs72833480 | A | G | 0.296 | 0.011 | 0.013 | 3.99E-01 | rs144843919 | A | G | 0.036 | -0.013 | 0.033 | 6.98E-01 |  |
| rs7575873 | G | A | 0.130 | -0.012 | 0.018 | 4.99E-01 | rs7575873 | G | A | 0.130 | -0.012 | 0.018 | 4.99E-01 |  |
| rs6040076 | G | C | 0.495 | -0.012 | 0.012 | 3.36E-01 | rs6040076 | G | C | 0.495 | -0.012 | 0.012 | 3.36E-01 |  |
| rs28530618 | A | G | 0.472 | 0.011 | 0.012 | 3.70E-01 | rs28530618 | A | G | 0.472 | 0.011 | 0.012 | 3.70E-01 |  |
| rs2229742 | C | G | 0.104 | 0.015 | 0.020 | 4.45E-01 | rs2229742 | C | G | 0.104 | 0.015 | 0.020 | 4.45E-01 |  |
| rs134594 | C | T | 0.352 | -0.004 | 0.013 | 7.50E-01 | rs134594 | C | T | 0.352 | -0.004 | 0.013 | 7.50E-01 |  |
| rs41311445 | C | A | 0.096 | -0.028 | 0.020 | 1.69E-01 | rs62240962 | T | C | 0.082 | -0.027 | 0.022 | 2.21E-01 |  |
| rs2168443 | T | A | 0.380 | 0.006 | 0.012 | 6.19E-01 | rs2242116 | A | G | 0.379 | 0.007 | 0.012 | 5.62E-01 |  |
| rs11719201 | T | C | 0.249 | 0.004 | 0.014 | 7.92E-01 | rs11719201 | T | C | 0.249 | 0.004 | 0.014 | 7.92E-01 |  |
| rs10935733 | T | C | 0.391 | 0.000 | 0.012 | 9.82E-01 | rs10935733 | T | C | 0.391 | 0.000 | 0.012 | 9.82E-01 |  |
| rs900399 | G | A | 0.397 | 0.004 | 0.012 | 7.34E-01 | rs13322435 | G | A | 0.401 | 0.005 | 0.012 | 7.02E-01 |  |
| rs4144829 | C | T | 0.263 | 0.011 | 0.014 | 4.23E-01 | rs925098 | G | A | 0.265 | 0.011 | 0.014 | 4.38E-01 |  |
| rs2946179 | T | C | 0.260 | -0.006 | 0.014 | 6.54E-01 | rs6537307 | G | A | 0.493 | 0.008 | 0.012 | 5.27E-01 |  |
| rs35261542 | A | C | 0.262 | 0.012 | 0.014 | 3.93E-01 | rs7729301 | G | A | 0.260 | -0.006 | 0.014 | 6.56E-01 |  |
| rs9368777 | G | C | 0.425 | -0.001 | 0.012 | 9.21E-01 | rs35261542 | A | C | 0.262 | 0.012 | 0.014 | 3.93E-01 |  |
| rs10872678 | C | T | 0.275 | 0.005 | 0.013 | 7.21E-01 | rs1415701 | A | G | 0.267 | -0.016 | 0.014 | 2.44E-01 |  |
| rs798498 | G | T | 0.307 | -0.002 | 0.013 | 8.82E-01 | rs1101081 | T | C | 0.275 | 0.004 | 0.013 | 7.44E-01 |  |
| rs11765649 | C | T | 0.261 | 0.008 | 0.014 | 5.58E-01 | rs798489 | T | C | 0.270 | 0.003 | 0.013 | 8.20E-01 |  |
| rs111778406 | G | A | 0.072 | 0.023 | 0.023 | 3.16E-01 | rs11765649 | C | T | 0.261 | 0.008 | 0.014 | 5.58E-01 |  |
| rs13266210 | G | A | 0.213 | 0.002 | 0.015 | 8.69E-01 | rs6959887 | G | A | 0.387 | -0.001 | 0.012 | 9.61E-01 |  |
| rs12543725 | A | G | 0.413 | 0.003 | 0.012 | 8.05E-01 | rs62466330 | C | T | 0.070 | 0.027 | 0.023 | 2.49E-01 |  |
| rs7854962 | G | C | 0.215 | 0.002 | 0.015 | 9.11E-01 | rs13266210 | G | A | 0.213 | 0.002 | 0.015 | 8.69E-01 |  |
| rs3780573 | A | G | 0.095 | -0.029 | 0.021 | 1.54E-01 | rs12543725 | A | G | 0.413 | 0.003 | 0.012 | 8.05E-01 |  |
| rs1411424 | G | A | 0.478 | 0.012 | 0.012 | 3.01E-01 | rs28510415 | G | A | 0.095 | -0.030 | 0.021 | 1.44E-01 |  |
| rs10818797 | C | T | 0.141 | -0.018 | 0.017 | 3.08E-01 | rs2150052 | A | T | 0.495 | 0.011 | 0.012 | 3.70E-01 |  |
|  |  |  |  |  |  |  | rs7847628 | A | G | 0.321 | 0.010 | 0.013 | 4.28 E-01 |  |

| **Supplementary Table 9. The genetic associations between the lower BW on SHBG.** | | | | | | | | | | | | | |
| --- | --- | --- | --- | --- | --- | --- | --- | --- | --- | --- | --- | --- | --- |
| **SNP (47)** | **EA** | **OA** | **EAF** | **β** | **SE** | **P-value** | **SNP (48)** | **EA** | **OA** | **EAF** | **β** | **SE** | **P-value** |
| rs2473248 | T | C | 0.118 | -0.001 | 0.002 | 8.10E-01 | rs2473248 | T | C | 0.118 | -0.001 | 0.002 | 8.10E-01 |
| rs3753639 | T | C | 0.755 | 0.005 | 0.002 | 8.30E-03 | rs3753639 | T | C | 0.755 | 0.005 | 0.002 | 8.30E-03 |
| rs72480273 | A | C | 0.810 | 0.003 | 0.002 | 3.00E-02 | rs72480273 | A | C | 0.810 | 0.003 | 0.002 | 3.00E-02 |
| rs7575873 | A | G | 0.871 | -0.001 | 0.002 | 6.60E-01 | rs7575873 | A | G | 0.871 | -0.001 | 0.002 | 6.60E-01 |
| rs17034876 | C | T | 0.302 | 0.002 | 0.002 | 1.40E-01 | rs1374204 | C | T | 0.302 | 0.002 | 0.002 | 1.30E-01 |
| rs2168443 | T | A | 0.381 | 0.008 | 0.001 | 3.20E-08 | rs2242116 | A | G | 0.380 | 0.008 | 0.001 | 4.50E-08 |
| rs11719201 | C | T | 0.752 | -0.007 | 0.002 | 7.70E-07 | rs11719201 | C | T | 0.752 | -0.007 | 0.002 | 7.70E-07 |
| rs10935733 | T | C | 0.391 | 0.003 | 0.001 | 2.20E-02 | rs10935733 | T | C | 0.391 | 0.003 | 0.001 | 2.20E-02 |
| rs900399 | A | G | 0.602 | 0.002 | 0.001 | 1.90E-01 | rs13322435 | A | G | 0.598 | 0.002 | 0.001 | 1.10E-01 |
| rs4144829 | C | T | 0.263 | 0.010 | 0.002 | 1.90E-08 | rs925098 | G | A | 0.265 | 0.010 | 0.002 | 7.60E-09 |
| rs2131354 | G | A | 0.472 | -0.004 | 0.001 | 1.80E-03 | rs6537307 | A | G | 0.505 | -0.003 | 0.001 | 4.30E-02 |
| rs854037 | A | G | 0.813 | 0.000 | 0.002 | 9.70E-01 | rs854037 | A | G | 0.813 | 0.000 | 0.002 | 9.70E-01 |
| rs2946179 | T | C | 0.260 | -0.008 | 0.002 | 1.70E-07 | rs7729301 | G | A | 0.260 | -0.008 | 0.002 | 1.50E-07 |
| rs35261542 | C | A | 0.738 | 0.009 | 0.002 | 6.00E-09 | rs35261542 | C | A | 0.738 | 0.009 | 0.002 | 6.00E-09 |
| rs9368777 | G | C | 0.426 | 0.001 | 0.001 | 5.40E-01 | rs7742369 | A | G | 0.825 | 0.000 | 0.002 | 7.70E-01 |
| rs1187118 | A | T | 0.168 | 0.000 | 0.002 | 7.70E-01 | rs1415701 | G | A | 0.732 | 0.007 | 0.002 | 6.30E-07 |
| rs10872678 | T | C | 0.725 | 0.001 | 0.002 | 2.00E-01 | rs1101081 | C | T | 0.725 | 0.001 | 0.002 | 2.00E-01 |
| rs798498 | T | G | 0.693 | 0.003 | 0.002 | 1.30E-01 | rs798489 | C | T | 0.730 | 0.002 | 0.002 | 3.00E-01 |
| rs11765649 | T | C | 0.739 | 0.004 | 0.002 | 3.50E-02 | rs11765649 | T | C | 0.739 | 0.004 | 0.002 | 3.50E-02 |
| rs138715366 | C | T | 0.991 | 0.012 | 0.008 | 1.80E-01 | rs6959887 | A | G | 0.613 | 0.001 | 0.001 | 6.40E-01 |
| rs111778406 | A | G | 0.928 | -0.007 | 0.003 | 1.40E-02 | rs138715366 | C | T | 0.991 | 0.012 | 0.008 | 1.80E-01 |
| rs13266210 | A | G | 0.786 | 0.001 | 0.002 | 6.00E-01 | rs62466330 | T | C | 0.930 | -0.008 | 0.003 | 6.90E-03 |
| rs12543725 | G | A | 0.587 | 0.003 | 0.001 | 6.80E-03 | rs13266210 | A | G | 0.786 | 0.001 | 0.002 | 6.00E-01 |
| rs7854962 | C | G | 0.785 | 0.005 | 0.002 | 2.60E-03 | rs12543725 | G | A | 0.587 | 0.003 | 0.001 | 6.80E-03 |
| rs3780573 | G | A | 0.904 | -0.003 | 0.002 | 1.80E-01 | rs28510415 | A | G | 0.905 | -0.003 | 0.002 | 1.70E-01 |
| rs1411424 | G | A | 0.477 | -0.002 | 0.001 | 1.20E-01 | rs2150052 | A | T | 0.495 | -0.001 | 0.001 | 3.10E-01 |
| rs10818797 | T | C | 0.859 | -0.009 | 0.002 | 8.50E-06 | rs7847628 | A | G | 0.321 | 0.007 | 0.002 | 1.90E-06 |
| rs2497304 | C | T | 0.521 | 0.000 | 0.001 | 5.40E-01 | rs700059 | G | A | 0.138 | 0.009 | 0.002 | 1.80E-06 |
| rs79237883 | T | C | 0.923 | -0.011 | 0.003 | 1.60E-05 | rs61862780 | T | C | 0.509 | 0.000 | 0.001 | 9.40E-01 |
| rs740746 | G | A | 0.266 | -0.003 | 0.002 | 1.20E-01 | rs7076938 | C | T | 0.265 | -0.003 | 0.002 | 1.20E-01 |
| rs72851023 | C | T | 0.924 | 0.002 | 0.003 | 6.40E-01 | rs2421016 | C | T | 0.529 | -0.003 | 0.001 | 5.60E-02 |
| rs2306547 | C | T | 0.531 | 0.000 | 0.001 | 7.10E-01 | rs72851023 | C | T | 0.924 | 0.002 | 0.003 | 6.40E-01 |
| rs7964361 | G | A | 0.910 | -0.003 | 0.002 | 1.30E-01 | rs10830963 | C | G | 0.726 | 0.006 | 0.002 | 2.40E-04 |
| rs7998537 | G | A | 0.680 | 0.001 | 0.002 | 2.20E-01 | rs139975827 | G | A | 0.627 | -0.003 | 0.002 | 1.10E-01 |
| rs1819436 | T | C | 0.124 | 0.002 | 0.002 | 3.70E-01 | rs12823128 | T | C | 0.530 | 0.000 | 0.001 | 7.80E-01 |
| rs12906125 | G | A | 0.674 | 0.003 | 0.002 | 3.90E-02 | rs1351394 | T | C | 0.491 | 0.008 | 0.001 | 3.10E-09 |
| rs7402982 | A | G | 0.415 | 0.006 | 0.001 | 4.50E-05 | rs7964361 | G | A | 0.910 | -0.003 | 0.002 | 1.30E-01 |
| rs28415607 | T | C | 0.754 | 0.001 | 0.002 | 6.20E-01 | rs1819436 | T | C | 0.124 | 0.002 | 0.002 | 3.70E-01 |
| rs113086489 | C | T | 0.455 | -0.008 | 0.001 | 2.60E-08 | rs12906125 | G | A | 0.674 | 0.003 | 0.002 | 3.90E-02 |
| rs144843919 | G | A | 0.963 | 0.004 | 0.004 | 3.30E-01 | rs7402982 | A | G | 0.415 | 0.006 | 0.001 | 4.50E-05 |
| rs72833480 | G | A | 0.705 | -0.001 | 0.002 | 5.10E-01 | rs113086489 | C | T | 0.455 | -0.008 | 0.001 | 2.60E-08 |
| rs6040076 | G | C | 0.496 | 0.001 | 0.001 | 6.00E-01 | rs144843919 | G | A | 0.963 | 0.004 | 0.004 | 3.30E-01 |
| rs28530618 | A | G | 0.473 | -0.003 | 0.001 | 5.70E-02 | rs6040076 | G | C | 0.496 | 0.001 | 0.001 | 6.00E-01 |
| rs6016377 | C | T | 0.551 | -0.004 | 0.001 | 2.30E-03 | rs28530618 | A | G | 0.473 | -0.003 | 0.001 | 5.70E-02 |
| rs2229742 | G | C | 0.896 | -0.003 | 0.002 | 2.10E-01 | rs6016377 | C | T | 0.551 | -0.004 | 0.001 | 2.30E-03 |
| rs134594 | C | T | 0.352 | 0.004 | 0.001 | 6.50E-03 | rs2229742 | G | C | 0.896 | -0.003 | 0.002 | 2.10E-01 |
| rs41311445 | A | C | 0.904 | 0.002 | 0.002 | 7.50E-01 | rs134594 | C | T | 0.352 | 0.004 | 0.001 | 6.50E-03 |
|  |  |  |  |  |  |  | rs62240962 | C | T | 0.918 | 0.002 | 0.003 | 6.90E-01 |

| **Supplementary Table 10. The genetic associations between the lower BW on Bio-T.** | | | | | | | | | | | | | |
| --- | --- | --- | --- | --- | --- | --- | --- | --- | --- | --- | --- | --- | --- |
| **SNP (47)** | **EA** | **OA** | **EAF** | **β** | **SE** | **P-value** | **SNP (48)** | **EA** | **OA** | **EAF** | **β** | **SE** | **P-value** |
| rs2473248 | T | C | 0.118 | 0.004 | 0.004 | 3.20E-01 | rs2473248 | T | C | 0.118 | 0.004 | 0.004 | 3.20E-01 |
| rs3753639 | T | C | 0.755 | 0.001 | 0.003 | 4.80E-01 | rs3753639 | T | C | 0.755 | 0.001 | 0.003 | 4.80E-01 |
| rs72480273 | A | C | 0.810 | -0.007 | 0.003 | 2.60E-02 | rs72480273 | A | C | 0.810 | -0.007 | 0.003 | 2.60E-02 |
| rs7575873 | A | G | 0.871 | -0.003 | 0.004 | 4.40E-01 | rs7575873 | A | G | 0.871 | -0.003 | 0.004 | 4.40E-01 |
| rs17034876 | C | T | 0.302 | -0.001 | 0.003 | 8.10E-01 | rs1374204 | C | T | 0.302 | 0.000 | 0.003 | 8.30E-01 |
| rs2168443 | T | A | 0.381 | -0.014 | 0.003 | 7.30E-08 | rs2242116 | A | G | 0.380 | -0.014 | 0.003 | 9.20E-08 |
| rs11719201 | C | T | 0.752 | 0.011 | 0.003 | 1.70E-04 | rs11719201 | C | T | 0.752 | 0.011 | 0.003 | 1.70E-04 |
| rs10935733 | T | C | 0.391 | -0.002 | 0.003 | 4.00E-01 | rs10935733 | T | C | 0.391 | -0.002 | 0.003 | 4.00E-01 |
| rs900399 | A | G | 0.602 | -0.011 | 0.003 | 3.70E-05 | rs13322435 | A | G | 0.598 | -0.012 | 0.003 | 7.20E-06 |
| rs4144829 | C | T | 0.263 | -0.016 | 0.003 | 2.30E-08 | rs925098 | G | A | 0.265 | -0.017 | 0.003 | 4.40E-09 |
| rs2131354 | G | A | 0.472 | 0.008 | 0.003 | 2.00E-03 | rs6537307 | A | G | 0.505 | 0.007 | 0.003 | 1.10E-02 |
| rs854037 | A | G | 0.813 | 0.001 | 0.003 | 7.40E-01 | rs854037 | A | G | 0.813 | 0.001 | 0.003 | 7.40E-01 |
| rs2946179 | T | C | 0.260 | 0.004 | 0.003 | 9.60E-02 | rs7729301 | G | A | 0.260 | 0.004 | 0.003 | 9.20E-02 |
| rs35261542 | C | A | 0.738 | -0.003 | 0.003 | 3.70E-01 | rs35261542 | C | A | 0.738 | -0.003 | 0.003 | 3.70E-01 |
| rs9368777 | G | C | 0.426 | -0.004 | 0.003 | 8.00E-02 | rs7742369 | A | G | 0.825 | 0.005 | 0.003 | 1.20E-01 |
| rs1187118 | A | T | 0.168 | -0.006 | 0.003 | 4.80E-02 | rs1415701 | G | A | 0.732 | -0.013 | 0.003 | 7.90E-06 |
| rs10872678 | T | C | 0.725 | 0.001 | 0.003 | 9.20E-01 | rs1101081 | C | T | 0.725 | 0.001 | 0.003 | 9.30E-01 |
| rs798498 | T | G | 0.693 | -0.006 | 0.003 | 3.30E-02 | rs798489 | C | T | 0.730 | -0.006 | 0.003 | 3.80E-02 |
| rs11765649 | T | C | 0.739 | -0.007 | 0.003 | 1.50E-02 | rs11765649 | T | C | 0.739 | -0.007 | 0.003 | 1.50E-02 |
| rs138715366 | C | T | 0.991 | -0.022 | 0.014 | 1.10E-01 | rs6959887 | A | G | 0.613 | 0.003 | 0.003 | 2.70E-01 |
| rs111778406 | A | G | 0.928 | 0.003 | 0.005 | 3.50E-01 | rs138715366 | C | T | 0.991 | -0.022 | 0.014 | 1.10E-01 |
| rs13266210 | A | G | 0.786 | 0.004 | 0.003 | 3.20E-01 | rs62466330 | T | C | 0.930 | 0.006 | 0.005 | 1.70E-01 |
| rs12543725 | G | A | 0.587 | -0.005 | 0.003 | 2.50E-02 | rs13266210 | A | G | 0.786 | 0.004 | 0.003 | 3.20E-01 |
| rs7854962 | C | G | 0.785 | -0.004 | 0.003 | 1.50E-01 | rs12543725 | G | A | 0.587 | -0.005 | 0.003 | 2.50E-02 |
| rs3780573 | G | A | 0.904 | 0.007 | 0.004 | 6.50E-02 | rs28510415 | A | G | 0.905 | 0.007 | 0.004 | 4.50E-02 |
| rs1411424 | G | A | 0.477 | 0.002 | 0.003 | 3.50E-01 | rs2150052 | A | T | 0.495 | 0.002 | 0.003 | 5.00E-01 |
| rs10818797 | T | C | 0.859 | 0.011 | 0.004 | 2.10E-03 | rs7847628 | A | G | 0.321 | -0.001 | 0.003 | 8.90E-01 |
| rs2497304 | C | T | 0.521 | 0.008 | 0.003 | 1.80E-03 | rs700059 | G | A | 0.138 | -0.011 | 0.004 | 2.10E-03 |
| rs79237883 | T | C | 0.923 | -0.005 | 0.005 | 3.80E-01 | rs61862780 | T | C | 0.509 | 0.009 | 0.003 | 1.80E-04 |
| rs740746 | G | A | 0.265 | 0.003 | 0.003 | 4.90E-01 | rs7076938 | C | T | 0.265 | 0.003 | 0.003 | 4.90E-01 |
| rs72851023 | C | T | 0.924 | 0.002 | 0.005 | 5.20E-01 | rs2421016 | C | T | 0.529 | 0.001 | 0.003 | 6.80E-01 |
| rs2306547 | C | T | 0.531 | 0.000 | 0.003 | 8.60E-01 | rs72851023 | C | T | 0.924 | 0.002 | 0.005 | 5.20E-01 |
| rs7964361 | G | A | 0.910 | 0.003 | 0.004 | 5.60E-01 | rs10830963 | C | G | 0.726 | -0.011 | 0.003 | 1.20E-04 |
| rs7998537 | G | A | 0.680 | -0.001 | 0.003 | 3.80E-01 | rs139975827 | G | A | 0.627 | 0.001 | 0.003 | 9.50E-01 |
| rs1819436 | T | C | 0.124 | 0.000 | 0.004 | 9.70E-01 | rs12823128 | T | C | 0.530 | 0.000 | 0.003 | 9.00E-01 |
| rs12906125 | G | A | 0.674 | -0.004 | 0.003 | 1.30E-01 | rs1351394 | T | C | 0.491 | -0.008 | 0.003 | 9.40E-04 |
| rs7402982 | A | G | 0.416 | -0.007 | 0.003 | 1.30E-02 | rs7964361 | G | A | 0.910 | 0.003 | 0.004 | 5.60E-01 |
| rs28415607 | T | C | 0.754 | 0.006 | 0.003 | 3.90E-02 | rs1819436 | T | C | 0.124 | 0.000 | 0.004 | 9.70E-01 |
| rs113086489 | C | T | 0.455 | 0.004 | 0.003 | 7.50E-02 | rs12906125 | G | A | 0.674 | -0.004 | 0.003 | 1.30E-01 |
| rs144843919 | G | A | 0.963 | 0.003 | 0.007 | 5.70E-01 | rs7402982 | A | G | 0.416 | -0.007 | 0.003 | 1.30E-02 |
| rs72833480 | G | A | 0.705 | 0.001 | 0.003 | 7.90E-01 | rs113086489 | C | T | 0.455 | 0.004 | 0.003 | 7.50E-02 |
| rs6040076 | G | C | 0.495 | 0.001 | 0.003 | 8.80E-01 | rs144843919 | G | A | 0.963 | 0.003 | 0.007 | 5.70E-01 |
| rs28530618 | A | G | 0.473 | 0.003 | 0.003 | 1.20E-01 | rs6040076 | G | C | 0.495 | 0.001 | 0.003 | 8.80E-01 |
| rs6016377 | C | T | 0.551 | -0.003 | 0.003 | 3.60E-01 | rs28530618 | A | G | 0.473 | 0.003 | 0.003 | 1.20E-01 |
| rs2229742 | G | C | 0.896 | 0.000 | 0.004 | 6.10E-01 | rs6016377 | C | T | 0.551 | -0.003 | 0.003 | 3.60E-01 |
| rs134594 | C | T | 0.352 | -0.004 | 0.003 | 2.00E-01 | rs2229742 | G | C | 0.896 | 0.000 | 0.004 | 6.10E-01 |
| rs41311445 | A | C | 0.904 | -0.011 | 0.004 | 9.10E-03 | rs134594 | C | T | 0.352 | -0.004 | 0.003 | 2.00E-01 |
|  |  |  |  |  |  |  | rs62240962 | C | T | 0.918 | -0.015 | 0.005 | 9.50E-04 |

| **Supplementary Table 11. The genetic associations between the lower BW on TT.** | | | | | | | | | | | | | |
| --- | --- | --- | --- | --- | --- | --- | --- | --- | --- | --- | --- | --- | --- |
| **SNP (47)** | **EA** | **OA** | **EAF** | **β** | **SE** | **P-value** | **SNP (48)** | **EA** | **OA** | **EAF** | **β** | **SE** | **P-value** |
| rs2473248 | T | C | 0.118 | 0.002 | 0.004 | 4.40E-01 | rs2473248 | T | C | 0.118 | 0.002 | 0.004 | 4.40E-01 |
| rs3753639 | T | C | 0.755 | 0.025 | 0.003 | 8.30E-16 | rs3753639 | T | C | 0.755 | 0.025 | 0.003 | 8.30E-16 |
| rs72480273 | A | C | 0.811 | -0.007 | 0.004 | 6.30E-02 | rs72480273 | A | C | 0.811 | -0.007 | 0.004 | 6.30E-02 |
| rs7575873 | A | G | 0.870 | -0.005 | 0.004 | 2.60E-01 | rs7575873 | A | G | 0.870 | -0.005 | 0.004 | 2.60E-01 |
| rs17034876 | C | T | 0.302 | 0.003 | 0.003 | 4.30E-01 | rs1374204 | C | T | 0.302 | 0.003 | 0.003 | 4.00E-01 |
| rs2168443 | T | A | 0.381 | -0.013 | 0.003 | 3.80E-05 | rs2242116 | A | G | 0.380 | -0.013 | 0.003 | 4.20E-05 |
| rs11719201 | C | T | 0.753 | 0.011 | 0.003 | 1.90E-03 | rs11719201 | C | T | 0.753 | 0.011 | 0.003 | 1.90E-03 |
| rs10935733 | T | C | 0.391 | 0.002 | 0.003 | 4.70E-01 | rs10935733 | T | C | 0.391 | 0.002 | 0.003 | 4.70E-01 |
| rs900399 | A | G | 0.602 | -0.014 | 0.003 | 1.60E-06 | rs13322435 | A | G | 0.598 | -0.013 | 0.003 | 3.60E-06 |
| rs4144829 | C | T | 0.262 | -0.006 | 0.003 | 2.10E-02 | rs925098 | G | A | 0.265 | -0.007 | 0.003 | 9.50E-03 |
| rs2131354 | G | A | 0.473 | -0.001 | 0.003 | 5.10E-01 | rs6537307 | A | G | 0.506 | 0.000 | 0.003 | 5.60E-01 |
| rs854037 | A | G | 0.813 | 0.000 | 0.004 | 7.90E-01 | rs854037 | A | G | 0.813 | 0.000 | 0.004 | 7.90E-01 |
| rs2946179 | T | C | 0.260 | -0.007 | 0.003 | 1.50E-02 | rs7729301 | G | A | 0.260 | -0.006 | 0.003 | 1.60E-02 |
| rs35261542 | C | A | 0.738 | 0.006 | 0.003 | 4.50E-02 | rs35261542 | C | A | 0.738 | 0.006 | 0.003 | 4.50E-02 |
| rs9368777 | G | C | 0.426 | -0.007 | 0.003 | 9.30E-03 | rs7742369 | A | G | 0.825 | 0.004 | 0.004 | 2.70E-01 |
| rs1187118 | A | T | 0.168 | -0.005 | 0.004 | 2.10E-01 | rs1415701 | G | A | 0.732 | -0.005 | 0.003 | 1.10E-01 |
| rs10872678 | T | C | 0.725 | 0.005 | 0.003 | 1.50E-01 | rs1101081 | C | T | 0.726 | 0.005 | 0.003 | 1.50E-01 |
| rs798498 | T | G | 0.693 | -0.009 | 0.003 | 3.90E-04 | rs798489 | C | T | 0.730 | -0.012 | 0.003 | 2.60E-05 |
| rs11765649 | T | C | 0.739 | -0.006 | 0.003 | 1.90E-02 | rs11765649 | T | C | 0.739 | -0.006 | 0.003 | 1.90E-02 |
| rs138715366 | C | T | 0.991 | -0.002 | 0.015 | 9.10E-01 | rs6959887 | A | G | 0.613 | 0.006 | 0.003 | 3.30E-02 |
| rs111778406 | A | G | 0.928 | -0.004 | 0.005 | 4.70E-01 | rs138715366 | C | T | 0.991 | -0.002 | 0.015 | 9.10E-01 |
| rs13266210 | A | G | 0.786 | 0.003 | 0.003 | 3.90E-01 | rs62466330 | T | C | 0.930 | -0.001 | 0.005 | 8.30E-01 |
| rs12543725 | G | A | 0.586 | -0.006 | 0.003 | 6.10E-02 | rs13266210 | A | G | 0.786 | 0.003 | 0.003 | 3.90E-01 |
| rs7854962 | C | G | 0.786 | 0.003 | 0.003 | 4.40E-01 | rs12543725 | G | A | 0.586 | -0.006 | 0.003 | 6.10E-02 |
| rs3780573 | G | A | 0.904 | 0.001 | 0.005 | 5.80E-01 | rs28510415 | A | G | 0.905 | 0.001 | 0.005 | 5.60E-01 |
| rs1411424 | G | A | 0.477 | 0.001 | 0.003 | 6.30E-01 | rs2150052 | A | T | 0.494 | 0.000 | 0.003 | 8.10E-01 |
| rs10818797 | T | C | 0.859 | -0.005 | 0.004 | 1.80E-01 | rs7847628 | A | G | 0.321 | 0.012 | 0.003 | 1.30E-04 |
| rs2497304 | C | T | 0.521 | 0.022 | 0.003 | 1.20E-14 | rs700059 | G | A | 0.138 | 0.006 | 0.004 | 1.10E-01 |
| rs79237883 | T | C | 0.923 | -0.030 | 0.005 | 6.10E-09 | rs61862780 | T | C | 0.509 | 0.024 | 0.003 | 8.20E-17 |
| rs740746 | G | A | 0.266 | 0.001 | 0.003 | 9.70E-01 | rs7076938 | C | T | 0.266 | 0.001 | 0.003 | 9.70E-01 |
| rs72851023 | C | T | 0.924 | 0.006 | 0.005 | 2.60E-01 | rs2421016 | C | T | 0.529 | -0.001 | 0.003 | 7.70E-01 |
| rs2306547 | C | T | 0.531 | -0.004 | 0.003 | 2.20E-01 | rs72851023 | C | T | 0.924 | 0.006 | 0.005 | 2.60E-01 |
| rs7964361 | G | A | 0.910 | -0.008 | 0.005 | 1.40E-01 | rs10830963 | C | G | 0.726 | -0.008 | 0.003 | 9.40E-03 |
| rs7998537 | G | A | 0.680 | -0.002 | 0.003 | 3.60E-01 | rs139975827 | G | A | 0.627 | -0.001 | 0.003 | 7.80E-01 |
| rs1819436 | T | C | 0.124 | 0.008 | 0.004 | 8.30E-02 | rs12823128 | T | C | 0.531 | -0.004 | 0.003 | 2.00E-01 |
| rs12906125 | G | A | 0.675 | -0.005 | 0.003 | 8.80E-02 | rs1351394 | T | C | 0.491 | 0.006 | 0.003 | 2.10E-02 |
| rs7402982 | A | G | 0.416 | -0.001 | 0.003 | 9.70E-01 | rs7964361 | G | A | 0.910 | -0.008 | 0.005 | 1.40E-01 |
| rs28415607 | T | C | 0.754 | 0.006 | 0.003 | 4.60E-02 | rs1819436 | T | C | 0.124 | 0.008 | 0.004 | 8.30E-02 |
| rs113086489 | C | T | 0.455 | -0.007 | 0.003 | 2.80E-02 | rs12906125 | G | A | 0.675 | -0.005 | 0.003 | 8.80E-02 |
| rs144843919 | G | A | 0.963 | 0.001 | 0.008 | 6.20E-01 | rs7402982 | A | G | 0.416 | -0.001 | 0.003 | 9.70E-01 |
| rs72833480 | G | A | 0.705 | -0.001 | 0.003 | 6.60E-01 | rs113086489 | C | T | 0.455 | -0.007 | 0.003 | 2.80E-02 |
| rs6040076 | G | C | 0.495 | 0.002 | 0.003 | 3.90E-01 | rs144843919 | G | A | 0.963 | 0.001 | 0.008 | 6.20E-01 |
| rs28530618 | A | G | 0.473 | 0.006 | 0.003 | 2.60E-02 | rs6040076 | G | C | 0.495 | 0.002 | 0.003 | 3.90E-01 |
| rs6016377 | C | T | 0.551 | -0.014 | 0.003 | 1.80E-05 | rs28530618 | A | G | 0.473 | 0.006 | 0.003 | 2.60E-02 |
| rs2229742 | G | C | 0.896 | -0.004 | 0.005 | 2.40E-01 | rs6016377 | C | T | 0.551 | -0.014 | 0.003 | 1.80E-05 |
| rs134594 | C | T | 0.351 | 0.000 | 0.003 | 9.90E-01 | rs2229742 | G | C | 0.896 | -0.004 | 0.005 | 2.40E-01 |
| rs41311445 | A | C | 0.904 | -0.016 | 0.005 | 6.10E-04 | rs134594 | C | T | 0.351 | 0.000 | 0.003 | 9.90E-01 |
|  |  |  |  |  |  |  | rs62240962 | C | T | 0.918 | -0.020 | 0.005 | 4.20E-05 |

| **Supplementary Table 12. The genetic associations between the lower BW on AMH.** | | | | | | | | | | | | | |
| --- | --- | --- | --- | --- | --- | --- | --- | --- | --- | --- | --- | --- | --- |
| **SNP (47)** | **EA** | **OA** | **EAF** | **β** | **SE** | **P-value** | **SNP (48)** | **EA** | **OA** | **EAF** | **β** | **SE** | **P-value** |
| 9:96900505 | C | G | 0.786 | -0.019 | 0.025 | 4.38E-01 | 5:157886953 | A | G | 0.734 | -0.009 | 0.022 | 7.01E-01 |
| 10:115792787 | A | G | 0.730 | 0.013 | 0.022 | 5.53E-01 | 3:46941116 | A | G | 0.388 | 0.031 | 0.021 | 1.46E-01 |
| 9:126020405 | T | C | 0.855 | -0.043 | 0.028 | 1.29E-01 | 7:44246271 | T | C | 0.011 | 0.029 | 0.114 | 7.99E-01 |
| 5:157886627 | T | C | 0.266 | 0.009 | 0.022 | 6.98E-01 | 4:145601863 | A | G | 0.495 | -0.002 | 0.020 | 9.20E-01 |
| 3:46947087 | A | T | 0.613 | -0.032 | 0.021 | 1.28E-01 | 8:142247979 | A | G | 0.423 | -0.003 | 0.020 | 8.68E-01 |
| 3:156798732 | A | G | 0.612 | 0.003 | 0.021 | 8.80E-01 | 7:73056805 | T | C | 0.931 | 0.032 | 0.041 | 4.30E-01 |
| 7:44246271 | T | C | 0.011 | 0.029 | 0.114 | 7.99E-01 | 7:35295365 | A | G | 0.609 | -0.032 | 0.021 | 1.21E-01 |
| 12:26877885 | T | C | 0.477 | 0.008 | 0.020 | 6.87E-01 | 10:124167512 | T | C | 0.468 | 0.004 | 0.020 | 8.44E-01 |
| 8:142247979 | A | G | 0.423 | -0.003 | 0.020 | 8.68E-01 | 20:39172728 | T | C | 0.441 | 0.005 | 0.021 | 7.94E-01 |
| 10:104940946 | T | C | 0.921 | 0.054 | 0.037 | 1.46E-01 | 8:41533514 | A | G | 0.776 | 0.021 | 0.024 | 3.94E-01 |
| 7:72957570 | A | G | 0.927 | 0.030 | 0.039 | 4.47E-01 | 12:102994878 | A | G | 0.090 | -0.017 | 0.035 | 6.24E-01 |
| 4:145599908 | A | G | 0.535 | -0.005 | 0.020 | 7.97E-01 | 3:156795468 | A | G | 0.603 | 0.003 | 0.021 | 8.81E-01 |
| 9:98239503 | A | G | 0.094 | -0.032 | 0.035 | 3.59E-01 | 2:46484205 | T | C | 0.702 | -0.009 | 0.022 | 6.77E-01 |
| 20:39172728 | T | C | 0.441 | 0.005 | 0.021 | 7.94E-01 | 3:148622968 | T | C | 0.394 | 0.000 | 0.021 | 9.99E-01 |
| 8:41533514 | A | G | 0.776 | 0.021 | 0.024 | 3.94E-01 | 12:66351826 | T | C | 0.496 | 0.009 | 0.020 | 6.68E-01 |
| 12:102994878 | A | G | 0.090 | -0.017 | 0.035 | 6.24E-01 | 11:2130620 | T | C | 0.077 | -0.018 | 0.038 | 6.39E-01 |
| 3:148622968 | T | C | 0.394 | 0.000 | 0.021 | 9.99E-01 | 22:42259524 | T | C | 0.091 | 0.017 | 0.036 | 6.31E-01 |
| 11:2130620 | T | C | 0.077 | -0.018 | 0.038 | 6.39E-01 | 20:31275581 | A | G | 0.468 | -0.012 | 0.020 | 5.58E-01 |
| 20:31275581 | A | G | 0.468 | -0.012 | 0.020 | 5.58E-01 | 9:125824055 | A | G | 0.860 | -0.054 | 0.029 | 5.87E-02 |
| 2:46484310 | T | C | 0.702 | -0.009 | 0.022 | 6.93E-01 | 4:17919811 | A | G | 0.744 | -0.004 | 0.023 | 8.71E-01 |
| 4:17903654 | T | C | 0.745 | -0.008 | 0.023 | 7.47E-01 | 22:29468456 | T | C | 0.662 | 0.007 | 0.021 | 7.49E-01 |
| 6:34169020 | A | T | 0.176 | -0.021 | 0.026 | 4.37E-01 | 21:16339172 | C | G | 0.109 | 0.067 | 0.033 | 4.10E-02 |
| 22:29468456 | T | C | 0.662 | 0.007 | 0.021 | 7.49E-01 | 11:92708710 | C | G | 0.724 | -0.017 | 0.022 | 4.63E-01 |
| 21:16339172 | C | G | 0.109 | 0.067 | 0.033 | 4.10E-02 | 6:34165721 | A | G | 0.816 | 0.013 | 0.026 | 6.23E-01 |
| 9:113892963 | A | G | 0.529 | 0.002 | 0.020 | 9.06E-01 | 9:113945067 | A | T | 0.493 | -0.011 | 0.020 | 6.02E-01 |
| 1:161644871 | A | C | 0.812 | -0.020 | 0.027 | 4.52E-01 | 1:161644871 | A | C | 0.812 | -0.020 | 0.027 | 4.52E-01 |
| 20:10658882 | C | G | 0.490 | -0.024 | 0.021 | 2.40E-01 | 20:10658882 | C | G | 0.490 | -0.024 | 0.021 | 2.40E-01 |
| 17:45964861 | A | G | 0.286 | 0.017 | 0.022 | 4.50E-01 | 1:22536643 | T | C | 0.118 | 0.014 | 0.032 | 6.66E-01 |
| 1:22536643 | T | C | 0.118 | 0.014 | 0.032 | 6.66E-01 | 13:78580283 | T | C | 0.125 | -0.026 | 0.031 | 3.90E-01 |
| 13:78580283 | T | C | 0.125 | -0.026 | 0.031 | 3.90E-01 | 17:7171356 | T | C | 0.556 | 0.008 | 0.021 | 7.01E-01 |
| 13:40662742 | A | G | 0.320 | -0.016 | 0.021 | 4.60E-01 | 10:94468643 | T | C | 0.501 | -0.020 | 0.020 | 3.27E-01 |
| 17:7171356 | T | C | 0.556 | 0.008 | 0.021 | 7.01E-01 | 3:123068744 | T | C | 0.250 | 0.001 | 0.023 | 9.67E-01 |
| 22:42070374 | A | C | 0.898 | -0.010 | 0.034 | 7.77E-01 | 17:29037339 | A | G | 0.041 | 0.114 | 0.058 | 4.75E-02 |
| 6:152039964 | T | C | 0.723 | 0.013 | 0.022 | 5.57E-01 | 6:130345835 | A | G | 0.270 | -0.027 | 0.023 | 2.33E-01 |
| 10:94492716 | T | C | 0.482 | 0.016 | 0.020 | 4.27E-01 | 6:152032917 | T | C | 0.276 | -0.013 | 0.022 | 5.47E-01 |
| 3:123068744 | T | C | 0.250 | 0.001 | 0.023 | 9.67E-01 | 10:115789375 | T | C | 0.730 | 0.014 | 0.022 | 5.20E-01 |
| 17:29037339 | A | G | 0.041 | 0.114 | 0.058 | 4.75E-02 | 1:154986091 | T | C | 0.767 | 0.036 | 0.024 | 1.31E-01 |
| 16:19993015 | T | C | 0.749 | -0.017 | 0.023 | 4.49E-01 | 5:57091783 | A | G | 0.824 | 0.031 | 0.027 | 2.48E-01 |
| 7:2795882 | T | G | 0.689 | 0.001 | 0.022 | 9.76E-01 | 7:2801803 | T | C | 0.275 | -0.006 | 0.022 | 8.05E-01 |
| 1:154986091 | T | C | 0.767 | 0.036 | 0.024 | 1.31E-01 | 12:26872730 | T | C | 0.523 | -0.009 | 0.020 | 6.63E-01 |
| 6:33788637 | C | G | 0.569 | 0.005 | 0.020 | 8.20E-01 | 6:20675792 | A | C | 0.263 | 0.000 | 0.023 | 9.95E-01 |
| 5:57091783 | A | G | 0.824 | 0.031 | 0.027 | 2.48E-01 | 9:98245026 | A | G | 0.908 | 0.036 | 0.035 | 2.98E-01 |
| 6:20675792 | A | C | 0.263 | 0.000 | 0.023 | 9.95E-01 | 5:157886953 | A | G | 0.734 | -0.009 | 0.022 | 7.01E-01 |

| **Supplementary Table 13. The genetic associations between the lower BW on menarche.** | | | | | | | | | | | | | |
| --- | --- | --- | --- | --- | --- | --- | --- | --- | --- | --- | --- | --- | --- |
| **SNP (47)** | **EA** | **OA** | **EAF** | **β** | **SE** | **P-value** | **SNP (48)** | **EA** | **OA** | **EAF** | **β** | **SE** | **P-value** |
| rs900399 | G | A | 0.399 | -0.016 | 0.002 | 5.50E-14 | rs2242116 | G | A | 0.618 | -0.001 | 0.002 | 6.40E-01 |
| rs740746 | A | G | 0.733 | -0.002 | 0.002 | 4.30E-01 | rs10830963 | G | C | 0.275 | -0.005 | 0.002 | 4.10E-02 |
| rs3780573 | A | G | 0.095 | 0.005 | 0.004 | 1.60E-01 | rs700059 | A | G | 0.862 | 0.002 | 0.003 | 4.90E-01 |
| rs9368777 | C | G | 0.574 | 0.003 | 0.002 | 1.20E-01 | rs7742369 | G | A | 0.175 | -0.004 | 0.003 | 1.60E-01 |
| rs12543725 | A | G | 0.414 | -0.003 | 0.002 | 1.60E-01 | rs12543725 | A | G | 0.414 | -0.003 | 0.002 | 1.60E-01 |
| rs144843919 | A | G | 0.037 | 0.006 | 0.006 | 3.00E-01 | rs144843919 | A | G | 0.037 | 0.006 | 0.006 | 3.00E-01 |
| rs1187118 | T | A | 0.832 | 0.003 | 0.003 | 2.20E-01 | rs1101081 | T | C | 0.273 | 0.000 | 0.002 | 8.60E-01 |
| rs10872678 | C | T | 0.274 | 0.000 | 0.002 | 8.50E-01 | rs7076938 | T | C | 0.733 | -0.002 | 0.002 | 4.10E-01 |
| rs798498 | G | T | 0.306 | -0.003 | 0.002 | 2.40E-01 | rs61862780 | C | T | 0.490 | -0.002 | 0.002 | 2.30E-01 |
| rs2946179 | C | T | 0.739 | 0.005 | 0.002 | 4.90E-02 | rs2150052 | T | A | 0.506 | 0.001 | 0.002 | 5.90E-01 |
| rs7998537 | A | G | 0.320 | -0.001 | 0.002 | 5.40E-01 | rs28510415 | G | A | 0.094 | 0.005 | 0.004 | 1.90E-01 |
| rs79237883 | C | T | 0.077 | 0.014 | 0.004 | 3.20E-04 | rs12823128 | C | T | 0.467 | 0.000 | 0.002 | 9.80E-01 |
| rs2131354 | A | G | 0.526 | 0.005 | 0.002 | 2.30E-02 | rs2473248 | C | T | 0.881 | 0.004 | 0.003 | 2.50E-01 |
| rs2306547 | T | C | 0.466 | 0.000 | 0.002 | 1.00E+00 | rs113086489 | T | C | 0.545 | 0.000 | 0.002 | 8.90E-01 |
| rs2473248 | C | T | 0.881 | 0.004 | 0.003 | 2.50E-01 | rs28530618 | G | A | 0.526 | 0.002 | 0.002 | 4.40E-01 |
| rs17034876 | T | C | 0.698 | -0.003 | 0.002 | 1.50E-01 | rs6016377 | T | C | 0.448 | 0.002 | 0.002 | 4.70E-01 |
| rs72833480 | A | G | 0.294 | 0.006 | 0.002 | 7.50E-03 | rs925098 | A | G | 0.736 | -0.007 | 0.002 | 1.70E-03 |
| rs113086489 | T | C | 0.545 | 0.000 | 0.002 | 8.90E-01 | rs72480273 | C | A | 0.187 | 0.007 | 0.003 | 9.30E-03 |
| rs28530618 | G | A | 0.526 | 0.002 | 0.002 | 4.40E-01 | rs3753639 | C | T | 0.244 | -0.001 | 0.002 | 7.60E-01 |
| rs6016377 | T | C | 0.448 | 0.002 | 0.002 | 4.70E-01 | rs798489 | T | C | 0.269 | -0.002 | 0.002 | 3.70E-01 |
| rs41311445 | C | A | 0.095 | 0.004 | 0.004 | 2.30E-01 | rs13266210 | G | A | 0.214 | -0.004 | 0.003 | 1.00E-01 |
| rs4144829 | T | C | 0.738 | -0.008 | 0.002 | 1.00E-03 | rs1351394 | C | T | 0.511 | -0.008 | 0.002 | 8.90E-05 |
| rs72480273 | C | A | 0.187 | 0.007 | 0.003 | 9.30E-03 | rs2229742 | C | G | 0.103 | -0.001 | 0.003 | 8.60E-01 |
| rs3753639 | C | T | 0.244 | -0.001 | 0.002 | 7.60E-01 | rs2421016 | T | C | 0.471 | 0.002 | 0.002 | 2.40E-01 |
| rs2497304 | T | C | 0.477 | -0.002 | 0.002 | 3.50E-01 | rs6537307 | G | A | 0.493 | 0.004 | 0.002 | 7.70E-02 |
| rs28415607 | C | T | 0.246 | -0.010 | 0.002 | 6.90E-05 | rs35261542 | A | C | 0.262 | 0.010 | 0.002 | 2.60E-05 |
| rs13266210 | G | A | 0.214 | -0.004 | 0.003 | 1.00E-01 | rs854037 | G | A | 0.186 | 0.000 | 0.003 | 8.60E-01 |
| rs2229742 | C | G | 0.103 | -0.001 | 0.003 | 8.60E-01 | rs11719201 | T | C | 0.246 | 0.004 | 0.002 | 8.70E-02 |
| rs7854962 | G | C | 0.214 | -0.003 | 0.003 | 1.80E-01 | rs62466330 | C | T | 0.069 | 0.003 | 0.004 | 5.00E-01 |
| rs35261542 | A | C | 0.262 | 0.010 | 0.002 | 2.60E-05 | rs72851023 | T | C | 0.075 | -0.002 | 0.004 | 6.40E-01 |
| rs854037 | G | A | 0.186 | 0.000 | 0.003 | 8.60E-01 | rs1819436 | C | T | 0.875 | 0.005 | 0.003 | 1.10E-01 |
| rs11719201 | T | C | 0.246 | 0.004 | 0.002 | 8.70E-02 | rs134594 | T | C | 0.650 | -0.006 | 0.002 | 6.70E-03 |
| rs1411424 | A | G | 0.523 | 0.003 | 0.002 | 1.60E-01 | rs1415701 | A | G | 0.267 | 0.000 | 0.002 | 9.70E-01 |
| rs72851023 | T | C | 0.075 | -0.002 | 0.004 | 6.40E-01 | rs138715366 | T | C | 0.009 | 0.003 | 0.011 | 7.80E-01 |
| rs1819436 | C | T | 0.875 | 0.005 | 0.003 | 1.10E-01 | rs7729301 | A | G | 0.739 | 0.005 | 0.002 | 4.70E-02 |
| rs134594 | T | C | 0.650 | -0.006 | 0.002 | 6.70E-03 | rs1374204 | T | C | 0.698 | -0.003 | 0.002 | 1.90E-01 |
| rs111778406 | G | A | 0.072 | 0.004 | 0.004 | 2.80E-01 | rs7964361 | A | G | 0.090 | 0.000 | 0.004 | 9.60E-01 |
| rs138715366 | T | C | 0.009 | 0.003 | 0.011 | 7.80E-01 | rs6040076 | C | G | 0.505 | 0.002 | 0.002 | 3.60E-01 |
| rs10818797 | C | T | 0.142 | -0.002 | 0.003 | 4.10E-01 | rs10935733 | C | T | 0.609 | -0.001 | 0.002 | 6.70E-01 |
| rs2168443 | A | T | 0.618 | -0.001 | 0.002 | 6.50E-01 | rs13322435 | G | A | 0.404 | -0.016 | 0.002 | 1.30E-14 |
| rs7964361 | A | G | 0.090 | 0.000 | 0.004 | 9.60E-01 | rs7575873 | G | A | 0.146 | -0.003 | 0.003 | 2.80E-01 |
| rs6040076 | C | G | 0.505 | 0.002 | 0.002 | 3.60E-01 | rs11765649 | C | T | 0.240 | 0.003 | 0.002 | 2.30E-01 |
| rs10935733 | C | T | 0.609 | -0.001 | 0.002 | 6.70E-01 | rs7402982 | G | A | 0.603 | 0.000 | 0.002 | 8.70E-01 |
| rs7575873 | G | A | 0.146 | -0.003 | 0.003 | 2.80E-01 | rs12906125 | A | G | 0.325 | 0.000 | 0.002 | 8.50E-01 |
| rs11765649 | C | T | 0.240 | 0.003 | 0.002 | 2.30E-01 | rs2242116 | G | A | 0.618 | -0.001 | 0.002 | 6.40E-01 |
| rs7402982 | G | A | 0.603 | 0.000 | 0.002 | 8.70E-01 | rs10830963 | G | C | 0.275 | -0.005 | 0.002 | 4.10E-02 |
| rs12906125 | A | G | 0.325 | 0.000 | 0.002 | 8.50E-01 | rs700059 | A | G | 0.862 | 0.002 | 0.003 | 4.90E-01 |

| **Supplementary Table 14. The genetic associations between the lower BW on menopause.** | | | | | | | | | | | | | |
| --- | --- | --- | --- | --- | --- | --- | --- | --- | --- | --- | --- | --- | --- |
| **SNP (47)** | **EA** | **OA** | **EAF** | **β** | **SE** | **P-value** | **SNP (48)** | **EA** | **OA** | **EAF** | **β** | **SE** | **P-value** |
| rs9368777 | C | G | 0.574 | 0.000 | 0.001 | 9.50E-01 | rs6537307 | G | A | 0.493 | -0.003 | 0.001 | 6.50E-02 |
| rs6016377 | T | C | 0.449 | -0.001 | 0.001 | 5.40E-01 | rs62466330 | C | T | 0.069 | -0.001 | 0.003 | 7.10E-01 |
| rs2229742 | C | G | 0.103 | 0.000 | 0.002 | 9.60E-01 | rs925098 | A | G | 0.735 | 0.000 | 0.002 | 8.00E-01 |
| rs7964361 | A | G | 0.089 | -0.001 | 0.002 | 8.00E-01 | rs1374204 | T | C | 0.698 | 0.001 | 0.002 | 5.50E-01 |
| rs138715366 | T | C | 0.009 | -0.006 | 0.007 | 4.30E-01 | rs28510415 | G | A | 0.094 | 0.003 | 0.002 | 1.90E-01 |
| rs17034876 | T | C | 0.698 | 0.001 | 0.002 | 5.10E-01 | rs6016377 | T | C | 0.449 | -0.001 | 0.001 | 5.40E-01 |
| rs10872678 | C | T | 0.273 | 0.003 | 0.002 | 3.80E-02 | rs2229742 | C | G | 0.103 | 0.000 | 0.002 | 9.60E-01 |
| rs798498 | G | T | 0.306 | -0.002 | 0.001 | 1.80E-01 | rs1101081 | T | C | 0.272 | 0.003 | 0.002 | 3.30E-02 |
| rs1187118 | T | A | 0.833 | 0.002 | 0.002 | 2.00E-01 | rs2421016 | T | C | 0.470 | -0.002 | 0.001 | 1.30E-01 |
| rs6040076 | C | G | 0.506 | -0.001 | 0.001 | 4.70E-01 | rs7076938 | T | C | 0.732 | -0.002 | 0.002 | 2.70E-01 |
| rs144843919 | A | G | 0.038 | -0.002 | 0.004 | 6.00E-01 | rs7964361 | A | G | 0.089 | -0.001 | 0.002 | 8.00E-01 |
| rs12543725 | A | G | 0.414 | 0.000 | 0.001 | 7.30E-01 | rs138715366 | T | C | 0.009 | -0.006 | 0.007 | 4.30E-01 |
| rs72851023 | T | C | 0.075 | 0.001 | 0.003 | 5.80E-01 | rs798489 | T | C | 0.270 | -0.001 | 0.002 | 4.90E-01 |
| rs28415607 | C | T | 0.245 | -0.003 | 0.002 | 7.80E-02 | rs6040076 | C | G | 0.506 | -0.001 | 0.001 | 4.70E-01 |
| rs113086489 | T | C | 0.545 | -0.004 | 0.001 | 8.30E-03 | rs144843919 | A | G | 0.038 | -0.002 | 0.004 | 6.00E-01 |
| rs2497304 | T | C | 0.478 | -0.001 | 0.001 | 6.60E-01 | rs12543725 | A | G | 0.414 | 0.000 | 0.001 | 7.30E-01 |
| rs2131354 | A | G | 0.526 | -0.003 | 0.001 | 2.90E-02 | rs72851023 | T | C | 0.075 | 0.001 | 0.003 | 5.80E-01 |
| rs7854962 | G | C | 0.214 | 0.002 | 0.002 | 1.50E-01 | rs113086489 | T | C | 0.545 | -0.004 | 0.001 | 8.30E-03 |
| rs3780573 | A | G | 0.095 | 0.003 | 0.002 | 2.30E-01 | rs7742369 | G | A | 0.175 | -0.003 | 0.002 | 1.60E-01 |
| rs2306547 | T | C | 0.467 | -0.001 | 0.001 | 5.00E-01 | rs13322435 | G | A | 0.405 | 0.002 | 0.001 | 1.20E-01 |
| rs1819436 | C | T | 0.875 | -0.001 | 0.002 | 7.20E-01 | rs700059 | A | G | 0.862 | 0.001 | 0.002 | 5.90E-01 |
| rs134594 | T | C | 0.650 | -0.001 | 0.001 | 6.30E-01 | rs12823128 | C | T | 0.467 | -0.001 | 0.001 | 5.20E-01 |
| rs3753639 | C | T | 0.244 | 0.001 | 0.002 | 4.10E-01 | rs10830963 | G | C | 0.274 | -0.002 | 0.002 | 1.80E-01 |
| rs2168443 | A | T | 0.618 | 0.000 | 0.001 | 8.40E-01 | rs1819436 | C | T | 0.875 | -0.001 | 0.002 | 7.20E-01 |
| rs1411424 | A | G | 0.523 | -0.001 | 0.001 | 6.20E-01 | rs134594 | T | C | 0.650 | -0.001 | 0.001 | 6.30E-01 |
| rs35261542 | A | C | 0.262 | 0.001 | 0.002 | 4.60E-01 | rs3753639 | C | T | 0.244 | 0.001 | 0.002 | 4.10E-01 |
| rs111778406 | G | A | 0.072 | -0.002 | 0.003 | 5.40E-01 | rs1415701 | A | G | 0.268 | 0.005 | 0.002 | 8.90E-04 |
| rs72833480 | A | G | 0.294 | 0.000 | 0.002 | 9.90E-01 | rs35261542 | A | C | 0.262 | 0.001 | 0.002 | 4.60E-01 |
| rs740746 | A | G | 0.732 | -0.002 | 0.002 | 2.60E-01 | rs61862780 | C | T | 0.490 | -0.001 | 0.001 | 6.80E-01 |
| rs79237883 | C | T | 0.077 | -0.008 | 0.003 | 2.10E-03 | rs13266210 | G | A | 0.215 | 0.001 | 0.002 | 7.00E-01 |
| rs41311445 | C | A | 0.095 | -0.002 | 0.002 | 4.40E-01 | rs10935733 | C | T | 0.608 | 0.000 | 0.001 | 7.30E-01 |
| rs10818797 | C | T | 0.142 | 0.000 | 0.002 | 8.40E-01 | rs28530618 | G | A | 0.526 | 0.001 | 0.001 | 4.80E-01 |
| rs13266210 | G | A | 0.215 | 0.001 | 0.002 | 7.00E-01 | rs2242116 | G | A | 0.618 | 0.000 | 0.001 | 8.70E-01 |
| rs10935733 | C | T | 0.608 | 0.000 | 0.001 | 7.30E-01 | rs2473248 | C | T | 0.882 | 0.001 | 0.002 | 5.30E-01 |
| rs28530618 | G | A | 0.526 | 0.001 | 0.001 | 4.80E-01 | rs72480273 | C | A | 0.187 | 0.000 | 0.002 | 8.80E-01 |
| rs2473248 | C | T | 0.882 | 0.001 | 0.002 | 5.30E-01 | rs7729301 | A | G | 0.739 | 0.001 | 0.002 | 6.90E-01 |
| rs72480273 | C | A | 0.187 | 0.000 | 0.002 | 8.80E-01 | rs11719201 | T | C | 0.247 | 0.005 | 0.002 | 2.50E-03 |
| rs2946179 | C | T | 0.739 | 0.001 | 0.002 | 7.00E-01 | rs854037 | G | A | 0.187 | -0.002 | 0.002 | 1.70E-01 |
| rs11719201 | T | C | 0.247 | 0.005 | 0.002 | 2.50E-03 | rs1351394 | C | T | 0.511 | -0.001 | 0.001 | 5.60E-01 |
| rs4144829 | T | C | 0.738 | 0.000 | 0.002 | 8.80E-01 | rs2150052 | T | A | 0.505 | -0.001 | 0.001 | 4.00E-01 |
| rs900399 | G | A | 0.399 | 0.002 | 0.001 | 1.20E-01 | rs7575873 | G | A | 0.146 | -0.001 | 0.002 | 7.80E-01 |
| rs854037 | G | A | 0.187 | -0.002 | 0.002 | 1.70E-01 | rs11765649 | C | T | 0.239 | 0.003 | 0.002 | 6.00E-02 |
| rs7998537 | A | G | 0.320 | -0.001 | 0.001 | 7.30E-01 | rs7402982 | G | A | 0.602 | -0.001 | 0.001 | 5.20E-01 |
| rs7575873 | G | A | 0.146 | -0.001 | 0.002 | 7.80E-01 | rs12906125 | A | G | 0.325 | 0.002 | 0.001 | 1.30E-01 |
| rs11765649 | C | T | 0.239 | 0.003 | 0.002 | 6.00E-02 |  |  |  |  |  |  |  |
| rs7402982 | G | A | 0.602 | -0.001 | 0.001 | 5.20E-01 |  |  |  |  |  |  |  |
| rs12906125 | A | G | 0.325 | 0.002 | 0.001 | 1.30E-01 |  |  |  |  |  |  |  |

| **Supplementary Table 15. The genetic associations between the lower BW on endometriosis.** | | | | | | | | | | | | | |
| --- | --- | --- | --- | --- | --- | --- | --- | --- | --- | --- | --- | --- | --- |
| **SNP (47)** | **EA** | **OA** | **EAF** | **β** | **SE** | **P-value** | **SNP (48)** | **EA** | **OA** | **EAF** | **β** | **SE** | **P-value** |
| rs2473248 | T | C | 0.819 | -0.008 | 0.025 | 7.65E-01 | rs2473248 | T | C | 0.819 | -0.008 | 0.025 | 7.65E-01 |
| rs3753639 | T | C | 0.207 | 0.028 | 0.024 | 2.49E-01 | rs3753639 | T | C | 0.207 | 0.028 | 0.024 | 2.49E-01 |
| rs72480273 | A | C | 0.163 | 0.012 | 0.026 | 6.39E-01 | rs72480273 | A | C | 0.163 | 0.012 | 0.026 | 6.39E-01 |
| rs7575873 | A | G | 0.094 | -0.013 | 0.033 | 6.91E-01 | rs7575873 | A | G | 0.094 | -0.013 | 0.033 | 6.91E-01 |
| rs17034876 | C | T | 0.728 | 0.001 | 0.022 | 9.53E-01 | rs1374204 | C | T | 0.728 | 0.000 | 0.023 | 9.90E-01 |
| rs2168443 | T | A | 0.611 | 0.007 | 0.020 | 7.20E-01 | rs2242116 | A | G | 0.612 | 0.007 | 0.020 | 7.18E-01 |
| rs11719201 | C | T | 0.176 | 0.036 | 0.026 | 1.55E-01 | rs11719201 | C | T | 0.176 | 0.036 | 0.026 | 1.55E-01 |
| rs10935733 | T | C | 0.602 | 0.009 | 0.020 | 6.39E-01 | rs10935733 | T | C | 0.602 | 0.009 | 0.020 | 6.39E-01 |
| rs900399 | A | G | 0.315 | 0.044 | 0.021 | 3.67E-02 | rs13322435 | A | G | 0.332 | 0.050 | 0.021 | 1.46E-02 |
| rs4144829 | C | T | 0.674 | 0.017 | 0.021 | 4.15E-01 | rs925098 | G | A | 0.669 | 0.010 | 0.021 | 6.43E-01 |
| rs2131354 | G | A | 0.526 | 0.024 | 0.020 | 2.27E-01 | rs6537307 | A | G | 0.514 | 0.019 | 0.020 | 3.27E-01 |
| rs854037 | A | G | 0.208 | -0.012 | 0.024 | 6.33E-01 | rs854037 | A | G | 0.208 | -0.012 | 0.024 | 6.33E-01 |
| rs2946179 | T | C | 0.788 | 0.025 | 0.024 | 3.04E-01 | rs7729301 | G | A | 0.788 | 0.025 | 0.024 | 3.04E-01 |
| rs35261542 | C | A | 0.330 | -0.003 | 0.021 | 8.97E-01 | rs35261542 | C | A | 0.330 | -0.003 | 0.021 | 8.97E-01 |
| rs9368777 | G | C | 0.664 | 0.006 | 0.021 | 7.94E-01 | rs7742369 | A | G | 0.144 | 0.104 | 0.028 | 2.20E-04 |
| rs1187118 | A | T | 0.861 | -0.105 | 0.029 | 2.51E-04 | rs1415701 | G | A | 0.248 | 0.001 | 0.023 | 9.66E-01 |
| rs10872678 | T | C | 0.350 | 0.116 | 0.020 | 1.18E-08 | rs1101081 | C | T | 0.349 | 0.115 | 0.020 | 1.33E-08 |
| rs798498 | T | G | 0.363 | -0.023 | 0.020 | 2.60E-01 | rs798489 | C | T | 0.342 | -0.026 | 0.021 | 2.08E-01 |
| rs138715366 | C | T | 0.003 | -0.161 | 0.196 | 4.12E-01 | rs6959887 | A | G | 0.392 | -0.013 | 0.020 | 5.15E-01 |
| rs111778406 | A | G | 0.051 | -0.076 | 0.044 | 8.44E-02 | rs138715366 | C | T | 0.003 | -0.161 | 0.196 | 4.12E-01 |
| rs13266210 | A | G | 0.205 | 0.011 | 0.024 | 6.53E-01 | rs62466330 | T | C | 0.049 | -0.085 | 0.045 | 6.12E-02 |
| rs12543725 | G | A | 0.410 | -0.005 | 0.020 | 8.17E-01 | rs13266210 | A | G | 0.205 | 0.011 | 0.024 | 6.53E-01 |
| rs7854962 | C | G | 0.187 | -0.034 | 0.025 | 1.73E-01 | rs12543725 | G | A | 0.410 | -0.005 | 0.020 | 8.17E-01 |
| rs3780573 | G | A | 0.105 | -0.069 | 0.032 | 2.86E-02 | rs28510415 | A | G | 0.105 | -0.070 | 0.032 | 2.83E-02 |
| rs1411424 | G | A | 0.505 | 0.009 | 0.019 | 6.33E-01 | rs2150052 | A | T | 0.464 | 0.000 | 0.018 | 9.86E-01 |
| rs10818797 | T | C | 0.167 | -0.012 | 0.026 | 6.34E-01 | rs700059 | G | A | 0.828 | 0.009 | 0.026 | 7.38E-01 |
| rs2497304 | C | T | 0.529 | 0.003 | 0.019 | 8.60E-01 | rs61862780 | T | C | 0.530 | 0.008 | 0.019 | 6.77E-01 |
| rs79237883 | T | C | 0.083 | -0.054 | 0.035 | 1.22E-01 | rs7076938 | C | T | 0.748 | -0.002 | 0.022 | 9.14E-01 |
| rs740746 | G | A | 0.746 | 0.000 | 0.023 | 9.93E-01 | rs2421016 | C | T | 0.623 | 0.002 | 0.020 | 9.07E-01 |
| rs72851023 | C | T | 0.072 | -0.022 | 0.038 | 5.64E-01 | rs72851023 | C | T | 0.072 | -0.022 | 0.038 | 5.64E-01 |
| rs2306547 | C | T | 0.443 | -0.027 | 0.020 | 1.73E-01 | rs10830963 | C | G | 0.358 | 0.008 | 0.020 | 6.97E-01 |
| rs7964361 | G | A | 0.077 | -0.021 | 0.036 | 5.56E-01 | rs12823128 | T | C | 0.436 | -0.034 | 0.020 | 8.08E-02 |
| rs7998537 | G | A | 0.315 | 0.008 | 0.021 | 7.00E-01 | rs1351394 | T | C | 0.478 | 0.011 | 0.020 | 5.70E-01 |
| rs1819436 | T | C | 0.876 | -0.025 | 0.029 | 4.06E-01 | rs7964361 | G | A | 0.077 | -0.021 | 0.036 | 5.56E-01 |
| rs12906125 | G | A | 0.271 | -0.037 | 0.022 | 9.12E-02 | rs1819436 | T | C | 0.876 | -0.025 | 0.029 | 4.06E-01 |
| rs7402982 | A | G | 0.597 | 0.030 | 0.020 | 1.35E-01 | rs12906125 | G | A | 0.271 | -0.037 | 0.022 | 9.12E-02 |
| rs28415607 | T | C | 0.305 | 0.069 | 0.021 | 1.12E-03 | rs7402982 | A | G | 0.597 | 0.030 | 0.020 | 1.35E-01 |
| rs113086489 | C | T | 0.600 | 0.006 | 0.020 | 7.61E-01 | rs113086489 | C | T | 0.600 | 0.006 | 0.020 | 7.61E-01 |
| rs144843919 | G | A | 0.021 | -0.161 | 0.069 | 1.92E-02 | rs144843919 | G | A | 0.021 | -0.161 | 0.069 | 1.92E-02 |
| rs72833480 | G | A | 0.294 | -0.033 | 0.021 | 1.22E-01 | rs6040076 | G | C | 0.430 | -0.013 | 0.020 | 5.13E-01 |
| rs6040076 | G | C | 0.430 | -0.013 | 0.020 | 5.13E-01 | rs28530618 | A | G | 0.483 | -0.005 | 0.019 | 8.00E-01 |
| rs28530618 | A | G | 0.483 | -0.005 | 0.019 | 8.00E-01 | rs6016377 | C | T | 0.363 | 0.004 | 0.020 | 8.37E-01 |
| rs6016377 | C | T | 0.363 | 0.004 | 0.020 | 8.37E-01 | rs2229742 | G | C | 0.111 | 0.079 | 0.031 | 1.09E-02 |
| rs2229742 | G | C | 0.111 | 0.079 | 0.031 | 1.09E-02 | rs134594 | C | T | 0.638 | -0.042 | 0.020 | 3.65E-02 |
| rs134594 | C | T | 0.638 | -0.042 | 0.020 | 3.65E-02 | rs62240962 | C | T | 0.105 | 0.037 | 0.032 | 2.41E-01 |
| rs41311445 | A | C | 0.134 | 0.033 | 0.029 | 2.43E-01 |  |  |  |  |  |  |  |

| **Supplementary Table 16. The genetic associations between the lower BW on leiomyoma.** | | | | | | | | | | | | | |
| --- | --- | --- | --- | --- | --- | --- | --- | --- | --- | --- | --- | --- | --- |
| **SNP (47)** | **EA** | **OA** | **EAF** | **β** | **SE** | **P-value** | **SNP (48)** | **EA** | **OA** | **EAF** | **β** | **SE** | **P-value** |
| rs2473248 | T | C | 0.818 | -0.016 | 0.019 | 3.92E-01 | rs2473248 | C | T | 0.818 | -0.016 | 0.019 | 3.92E-01 |
| rs3753639 | T | C | 0.208 | 0.058 | 0.018 | 1.26E-03 | rs3753639 | C | T | 0.208 | 0.058 | 0.018 | 1.26E-03 |
| rs72480273 | A | C | 0.163 | 0.010 | 0.020 | 6.25E-01 | rs72480273 | C | A | 0.163 | 0.010 | 0.020 | 6.25E-01 |
| rs7575873 | A | G | 0.094 | -0.001 | 0.025 | 9.64E-01 | rs7575873 | G | A | 0.094 | -0.001 | 0.025 | 9.64E-01 |
| rs17034876 | C | T | 0.728 | 0.001 | 0.017 | 9.62E-01 | rs1374204 | T | C | 0.728 | 0.000 | *NA* | 1.00E+00 |
| rs2168443 | T | A | 0.612 | 0.009 | 0.015 | 5.54E-01 | rs2242116 | G | A | 0.613 | 0.008 | 0.015 | 5.75E-01 |
| rs11719201 | C | T | 0.176 | 0.003 | 0.019 | 8.61E-01 | rs11719201 | T | C | 0.176 | 0.003 | 0.019 | 8.61E-01 |
| rs10935733 | T | C | 0.602 | 0.006 | 0.015 | 6.77E-01 | rs10935733 | C | T | 0.602 | 0.006 | 0.015 | 6.77E-01 |
| rs900399 | A | G | 0.315 | -0.010 | 0.016 | 5.35E-01 | rs13322435 | G | A | 0.332 | -0.013 | 0.015 | 3.93E-01 |
| rs4144829 | C | T | 0.675 | -0.017 | 0.015 | 2.60E-01 | rs925098 | A | G | 0.670 | -0.020 | 0.015 | 2.07E-01 |
| rs2131354 | G | A | 0.527 | 0.000 | 0.017 | 9.86E-01 | rs6537307 | G | A | 0.516 | 0.000 | 0.017 | 9.91E-01 |
| rs854037 | A | G | 0.208 | -0.001 | 0.017 | 9.53E-01 | rs854037 | G | A | 0.208 | -0.001 | 0.017 | 9.53E-01 |
| rs2946179 | T | C | 0.788 | 0.018 | 0.018 | 3.11E-01 | rs7729301 | A | G | 0.788 | 0.018 | 0.018 | 3.11E-01 |
| rs35261542 | C | A | 0.330 | -0.004 | 0.015 | 7.99E-01 | rs35261542 | A | C | 0.330 | -0.004 | 0.015 | 7.99E-01 |
| rs9368777 | G | C | 0.663 | 0.000 | 0.017 | 9.81E-01 | rs7742369 | G | A | 0.145 | 0.064 | 0.021 | 2.11E-03 |
| rs1187118 | A | T | 0.860 | -0.066 | 0.021 | 1.87E-03 | rs1415701 | A | G | 0.247 | -0.029 | 0.017 | 9.09E-02 |
| rs10872678 | T | C | 0.351 | 0.023 | 0.015 | 1.28E-01 | rs1101081 | T | C | 0.349 | 0.026 | 0.015 | 8.81E-02 |
| rs798498 | T | G | 0.361 | -0.016 | 0.015 | 2.78E-01 | rs798489 | T | C | 0.341 | -0.018 | 0.015 | 2.30E-01 |
| rs138715366 | C | T | 0.003 | -0.100 | 0.152 | 5.11E-01 | rs6959887 | G | A | 0.393 | -0.003 | 0.015 | 8.42E-01 |
| rs111778406 | A | G | 0.051 | -0.035 | 0.033 | 2.85E-01 | rs138715366 | T | C | 0.003 | -0.100 | 0.152 | 5.11E-01 |
| rs13266210 | A | G | 0.204 | -0.019 | 0.018 | 2.91E-01 | rs62466330 | C | T | 0.048 | -0.039 | 0.034 | 2.45E-01 |
| rs12543725 | G | A | 0.410 | -0.016 | 0.015 | 2.78E-01 | rs13266210 | G | A | 0.204 | -0.019 | 0.018 | 2.91E-01 |
| rs7854962 | C | G | 0.189 | 0.023 | 0.019 | 2.23E-01 | rs12543725 | A | G | 0.410 | -0.016 | 0.015 | 2.78E-01 |
| rs3780573 | G | A | 0.105 | -0.053 | 0.024 | 2.61E-02 | rs28510415 | G | A | 0.105 | -0.053 | 0.024 | 2.57E-02 |
| rs1411424 | G | A | 0.506 | -0.001 | 0.015 | 9.30E-01 | rs2150052 | T | A | 0.464 | -0.007 | 0.014 | 6.13E-01 |
| rs10818797 | T | C | 0.167 | -0.024 | 0.019 | 2.24E-01 | rs700059 | A | G | 0.829 | 0.023 | 0.019 | 2.30E-01 |
| rs2497304 | C | T | 0.529 | 0.010 | 0.014 | 5.03E-01 | rs61862780 | C | T | 0.530 | 0.015 | 0.014 | 3.17E-01 |
| rs79237883 | T | C | 0.083 | 0.011 | 0.026 | 6.75E-01 | rs7076938 | T | C | 0.749 | 0.026 | 0.017 | 1.20E-01 |
| rs740746 | G | A | 0.747 | 0.026 | 0.017 | 1.23E-01 | rs2421016 | T | C | 0.624 | 0.033 | 0.015 | 2.58E-02 |
| rs72851023 | C | T | 0.072 | 0.025 | 0.028 | 3.76E-01 | rs72851023 | T | C | 0.072 | 0.025 | 0.028 | 3.76E-01 |
| rs2306547 | C | T | 0.442 | -0.005 | 0.015 | 7.23E-01 | rs10830963 | G | C | 0.358 | 0.025 | 0.015 | 1.03E-01 |
| rs7964361 | G | A | 0.078 | 0.054 | 0.027 | 4.72E-02 | rs12823128 | C | T | 0.435 | -0.003 | 0.014 | 8.19E-01 |
| rs7998537 | G | A | 0.315 | 0.013 | 0.016 | 4.02E-01 | rs1351394 | C | T | 0.478 | 0.002 | 0.015 | 8.71E-01 |
| rs1819436 | T | C | 0.875 | 0.023 | 0.022 | 2.92E-01 | rs7964361 | A | G | 0.078 | 0.054 | 0.027 | 4.72E-02 |
| rs12906125 | G | A | 0.272 | 0.030 | 0.016 | 6.23E-02 | rs1819436 | C | T | 0.875 | 0.023 | 0.022 | 2.92E-01 |
| rs7402982 | A | G | 0.597 | -0.029 | 0.015 | 5.44E-02 | rs12906125 | A | G | 0.272 | 0.030 | 0.016 | 6.23E-02 |
| rs28415607 | T | C | 0.306 | 0.026 | 0.016 | 1.00E-01 | rs7402982 | G | A | 0.597 | -0.029 | 0.015 | 5.44E-02 |
| rs113086489 | C | T | 0.600 | 0.044 | 0.015 | 3.01E-03 | rs113086489 | T | C | 0.600 | 0.044 | 0.015 | 3.01E-03 |
| rs144843919 | G | A | 0.021 | -0.060 | 0.051 | 2.42E-01 | rs144843919 | A | G | 0.021 | -0.060 | 0.051 | 2.42E-01 |
| rs72833480 | G | A | 0.293 | -0.001 | 0.016 | 9.66E-01 | rs6040076 | C | G | 0.430 | -0.028 | 0.015 | 5.47E-02 |
| rs6040076 | G | C | 0.430 | -0.028 | 0.015 | 5.47E-02 | rs28530618 | G | A | 0.482 | 0.009 | 0.014 | 5.16E-01 |
| rs28530618 | A | G | 0.482 | 0.009 | 0.014 | 5.16E-01 | rs6016377 | T | C | 0.361 | -0.004 | 0.015 | 7.83E-01 |
| rs6016377 | C | T | 0.361 | -0.004 | 0.015 | 7.83E-01 | rs2229742 | C | G | 0.110 | -0.001 | 0.024 | 9.83E-01 |
| rs2229742 | G | C | 0.110 | -0.001 | 0.024 | 9.83E-01 | rs134594 | T | C | 0.641 | 0.029 | 0.015 | 5.61E-02 |
| rs134594 | C | T | 0.641 | 0.029 | 0.015 | 5.61E-02 | rs62240962 | T | C | 0.104 | -0.059 | 0.024 | 1.29E-02 |
| rs41311445 | A | C | 0.134 | -0.050 | 0.021 | 2.03E-02 |  |  |  |  |  |  |  |

| **Supplementary Table 17. The genetic associations between the lower BW on PCOS.** | | | | | | | | | | | | | |
| --- | --- | --- | --- | --- | --- | --- | --- | --- | --- | --- | --- | --- | --- |
| **SNP (47)** | **EA** | **OA** | **EAF** | **β** | **SE** | **P-value** | **SNP (48)** | **EA** | **OA** | **EAF** | **β** | **SE** | **P-value** |
| 10:94492716 | T | C | 0.470 | -0.100 | 0.031 | 1.30E-03 | 4:145601863 | A | G | 0.500 | 0.083 | 0.031 | 8.30E-03 |
| 4:145599908 | A | G | 0.530 | -0.087 | 0.031 | 5.50E-03 | 10:94468643 | T | C | 0.510 | 0.079 | 0.031 | 1.10E-02 |
| 7:72957570 | A | G | 0.930 | 0.160 | 0.063 | 9.00E-03 | 12:102994878 | A | G | 0.089 | -0.130 | 0.055 | 1.70E-02 |
| 12:102994878 | A | G | 0.089 | -0.130 | 0.055 | 1.70E-02 | 7:73056805 | T | C | 0.930 | 0.140 | 0.062 | 2.80E-02 |
| 9:96900505 | C | G | 0.790 | -0.092 | 0.039 | 1.80E-02 | 6:152032917 | T | C | 0.290 | 0.070 | 0.034 | 4.20E-02 |
| 6:152039964 | T | C | 0.710 | -0.067 | 0.035 | 5.20E-02 | 6:130345835 | A | G | 0.270 | -0.067 | 0.037 | 6.70E-02 |
| 6:33788637 | C | G | 0.580 | -0.059 | 0.031 | 6.20E-02 | 3:148622968 | T | C | 0.430 | -0.054 | 0.032 | 9.20E-02 |
| 3:148622968 | T | C | 0.430 | -0.054 | 0.032 | 9.20E-02 | 9:125824055 | A | G | 0.850 | -0.073 | 0.044 | 9.60E-02 |
| 3:46947087 | A | T | 0.640 | 0.053 | 0.033 | 1.00E-01 | 3:46941116 | A | G | 0.360 | -0.053 | 0.033 | 1.00E-01 |
| 1:154986091 | T | C | 0.750 | -0.059 | 0.037 | 1.10E-01 | 1:154986091 | T | C | 0.750 | -0.059 | 0.037 | 1.10E-01 |
| 9:126020405 | T | C | 0.850 | -0.068 | 0.043 | 1.20E-01 | 22:29468456 | T | C | 0.660 | 0.051 | 0.033 | 1.20E-01 |
| 22:29468456 | T | C | 0.660 | 0.051 | 0.033 | 1.20E-01 | 1:161644871 | A | C | 0.820 | -0.057 | 0.042 | 1.70E-01 |
| 1:161644871 | A | C | 0.820 | -0.057 | 0.042 | 1.70E-01 | 10:124167512 | T | C | 0.500 | -0.037 | 0.031 | 2.30E-01 |
| 17:7171356 | T | C | 0.550 | 0.038 | 0.032 | 2.30E-01 | 17:7171356 | T | C | 0.550 | 0.038 | 0.032 | 2.30E-01 |
| 10:115792787 | A | G | 0.750 | 0.041 | 0.036 | 2.50E-01 | 21:16339172 | C | G | 0.100 | 0.063 | 0.055 | 2.50E-01 |
| 21:16339172 | C | G | 0.100 | 0.063 | 0.055 | 2.50E-01 | 11:2130620 | T | C | 0.070 | 0.074 | 0.064 | 2.50E-01 |
| 11:2130620 | T | C | 0.070 | 0.074 | 0.064 | 2.50E-01 | 7:23479013 | T | C | 0.760 | 0.041 | 0.037 | 2.70E-01 |
| 13:40662742 | A | G | 0.320 | 0.037 | 0.033 | 2.60E-01 | 10:115789375 | T | C | 0.750 | 0.039 | 0.036 | 2.80E-01 |
| 7:23479013 | T | C | 0.760 | 0.041 | 0.037 | 2.70E-01 | 22:42259524 | T | C | 0.089 | 0.060 | 0.060 | 3.10E-01 |
| 9:98239503 | A | G | 0.110 | -0.053 | 0.050 | 2.90E-01 | 20:10658882 | C | G | 0.500 | 0.034 | 0.033 | 3.10E-01 |
| 20:10658882 | C | G | 0.500 | 0.034 | 0.033 | 3.10E-01 | 9:98245026 | A | G | 0.890 | 0.050 | 0.051 | 3.20E-01 |
| 15:99193269 | A | G | 0.430 | -0.031 | 0.034 | 3.60E-01 | 7:2801803 | T | C | 0.250 | -0.034 | 0.037 | 3.50E-01 |
| 6:34169020 | A | T | 0.160 | -0.035 | 0.043 | 4.20E-01 | 15:99193269 | A | G | 0.430 | -0.031 | 0.034 | 3.60E-01 |
| 22:42070374 | A | C | 0.890 | -0.042 | 0.056 | 4.60E-01 | 6:34165721 | A | G | 0.830 | 0.037 | 0.042 | 3.80E-01 |
| 8:41533514 | A | G | 0.800 | 0.028 | 0.039 | 4.80E-01 | 9:123631225 | A | G | 0.300 | -0.030 | 0.035 | 3.80E-01 |
| 5:157886627 | T | C | 0.270 | -0.023 | 0.035 | 5.00E-01 | 8:41533514 | A | G | 0.800 | 0.028 | 0.039 | 4.80E-01 |
| 13:78580283 | T | C | 0.140 | 0.029 | 0.048 | 5.40E-01 | 13:78580283 | T | C | 0.140 | 0.029 | 0.048 | 5.40E-01 |
| 20:39172728 | T | C | 0.410 | -0.019 | 0.033 | 5.70E-01 | 9:113945067 | A | T | 0.470 | -0.018 | 0.031 | 5.60E-01 |
| 5:57091783 | A | G | 0.810 | 0.020 | 0.039 | 6.00E-01 | 20:39172728 | T | C | 0.410 | -0.019 | 0.033 | 5.70E-01 |
| 10:104940946 | T | C | 0.920 | 0.028 | 0.056 | 6.10E-01 | 5:57091783 | A | G | 0.810 | 0.020 | 0.039 | 6.00E-01 |
| 1:22536643 | T | C | 0.110 | 0.024 | 0.050 | 6.40E-01 | 5:157886953 | A | G | 0.730 | 0.018 | 0.035 | 6.10E-01 |
| 4:17903654 | T | C | 0.730 | 0.015 | 0.035 | 6.60E-01 | 1:22536643 | T | C | 0.110 | 0.024 | 0.050 | 6.40E-01 |
| 17:45964861 | A | G | 0.290 | -0.015 | 0.034 | 6.70E-01 | 12:66351826 | T | C | 0.500 | -0.014 | 0.031 | 6.40E-01 |
| 6:20675792 | A | C | 0.260 | 0.012 | 0.035 | 7.20E-01 | 12:26872730 | T | C | 0.500 | -0.014 | 0.031 | 6.60E-01 |
| 12:26877885 | T | C | 0.510 | 0.011 | 0.031 | 7.30E-01 | 7:35295365 | A | G | 0.600 | 0.014 | 0.032 | 6.70E-01 |
| 17:29037339 | A | G | 0.045 | -0.026 | 0.088 | 7.70E-01 | 6:20675792 | A | C | 0.260 | 0.012 | 0.035 | 7.20E-01 |
| 20:31275581 | A | G | 0.470 | -0.005 | 0.031 | 8.70E-01 | 17:29037339 | A | G | 0.045 | -0.026 | 0.088 | 7.70E-01 |
| 3:123068744 | T | C | 0.220 | 0.005 | 0.038 | 8.90E-01 | 4:17919811 | A | G | 0.730 | 0.010 | 0.035 | 7.80E-01 |
| 16:19993015 | T | C | 0.730 | 0.005 | 0.036 | 8.90E-01 | 11:92708710 | C | G | 0.740 | -0.010 | 0.037 | 7.90E-01 |
| 2:46484310 | T | C | 0.700 | 0.004 | 0.037 | 9.10E-01 | 20:31275581 | A | G | 0.470 | -0.005 | 0.031 | 8.70E-01 |
| 7:2795882 | T | G | 0.710 | -0.004 | 0.034 | 9.10E-01 | 3:156795468 | A | G | 0.600 | 0.005 | 0.034 | 8.80E-01 |
| 8:142247979 | A | G | 0.420 | -0.003 | 0.032 | 9.20E-01 | 2:46484205 | T | C | 0.710 | 0.005 | 0.037 | 8.90E-01 |
| 9:113892963 | A | G | 0.540 | -0.003 | 0.031 | 9.30E-01 | 3:123068744 | T | C | 0.220 | 0.005 | 0.038 | 8.90E-01 |
| 3:156798732 | A | G | 0.620 | -0.003 | 0.033 | 9.40E-01 | 8:142247979 | A | G | 0.420 | -0.003 | 0.032 | 9.20E-01 |
| 2:23962647 | A | G | 0.880 | 0.001 | 0.048 | 9.80E-01 | 12:22068161 | A | G | 0.400 | -0.003 | 0.034 | 9.20E-01 |
| 15:91427612 | A | G | 0.330 | -0.001 | 0.036 | 9.90E-01 | 2:23962647 | A | G | 0.880 | 0.001 | 0.048 | 9.80E-01 |
|  |  |  |  |  |  |  | 15:91427612 | A | G | 0.330 | -0.001 | 0.036 | 9.90E-01 |


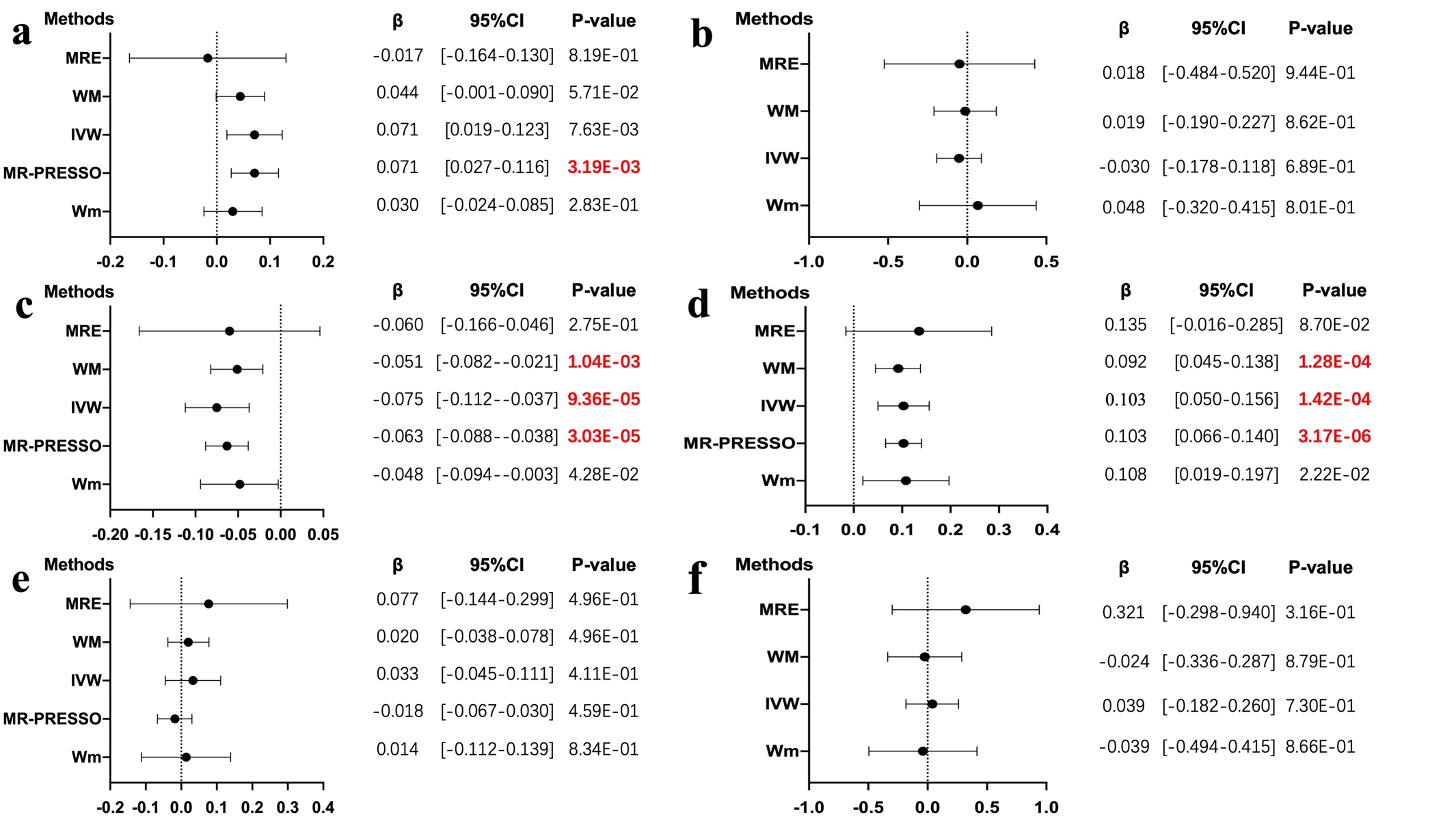
**Supplementary Figure 1. The results of four different methods of MR analysis.**


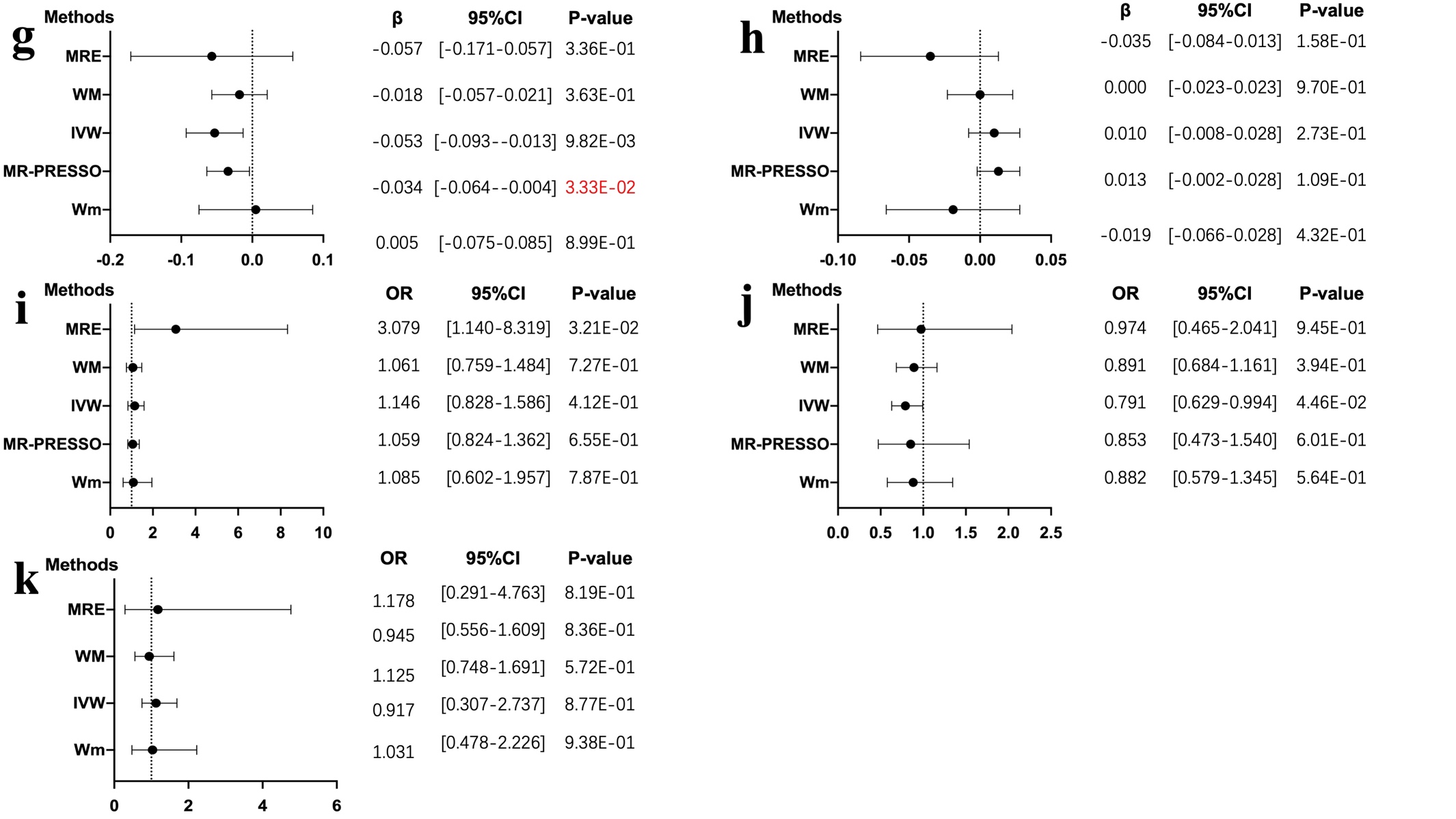
**Supplementary Figure 1. continued.**

**
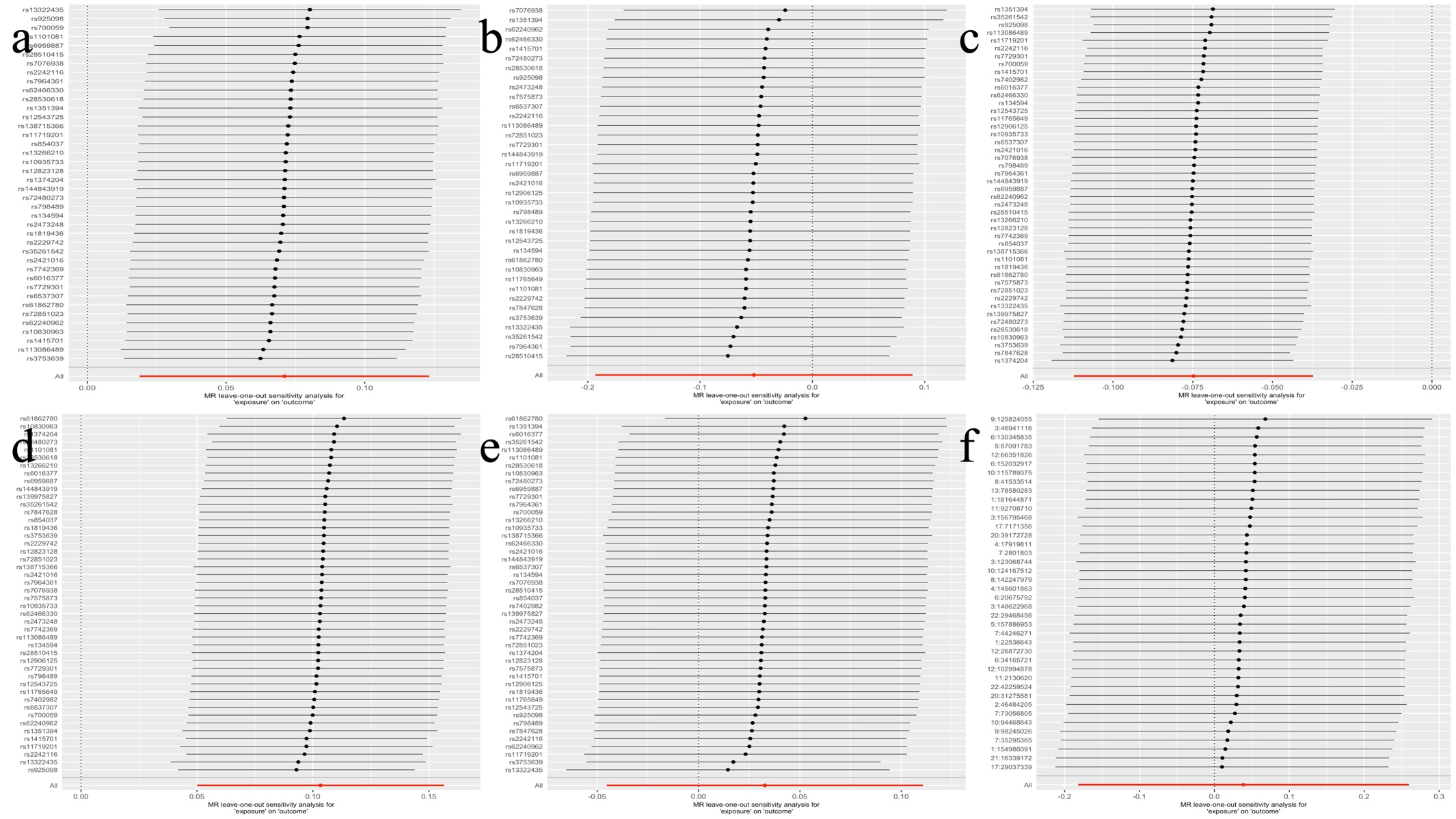
Supplementary Figure 2. The plot of leave-one-out analysis.**


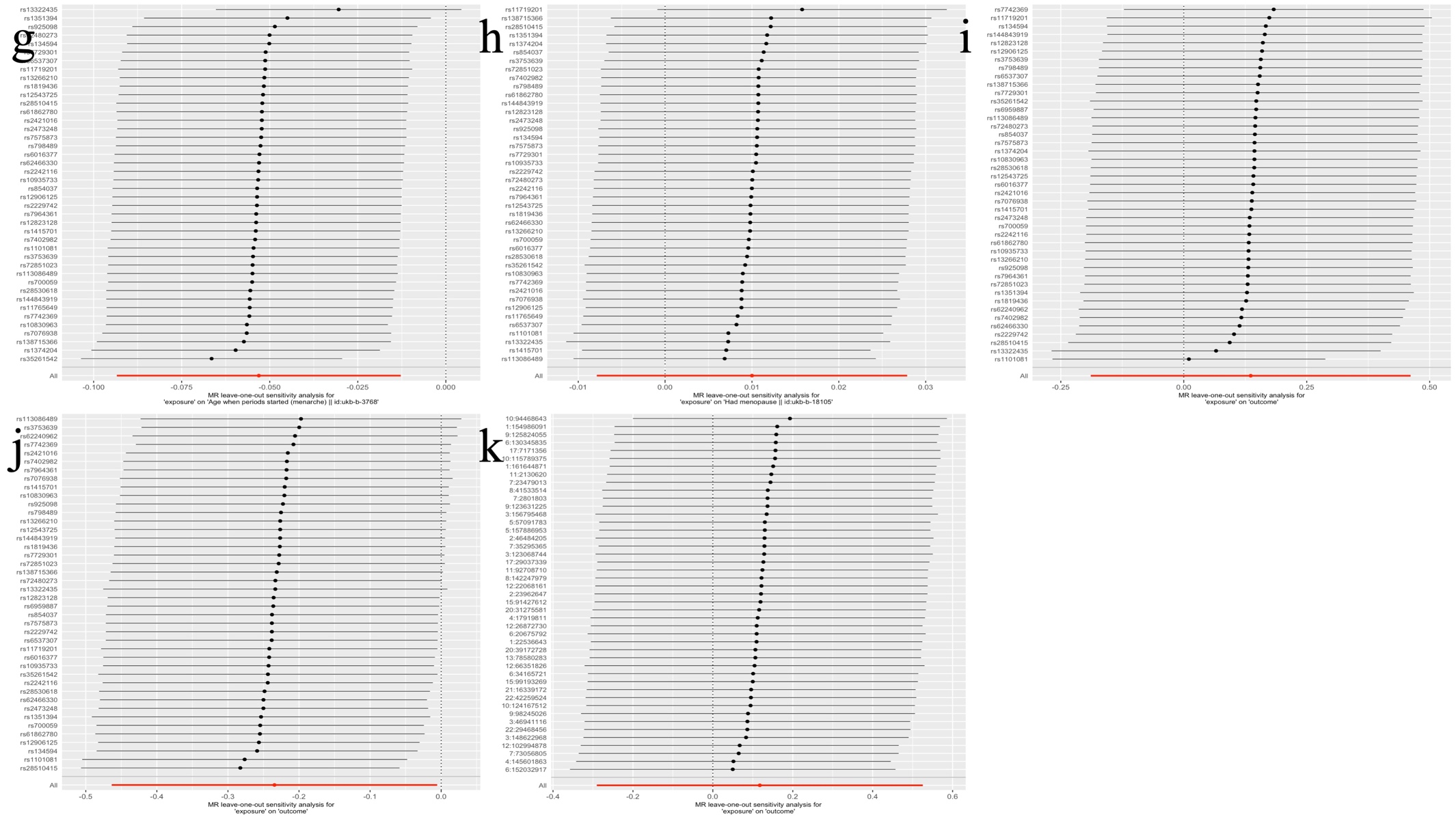
**Supplementary Figure 2. continued.**

**
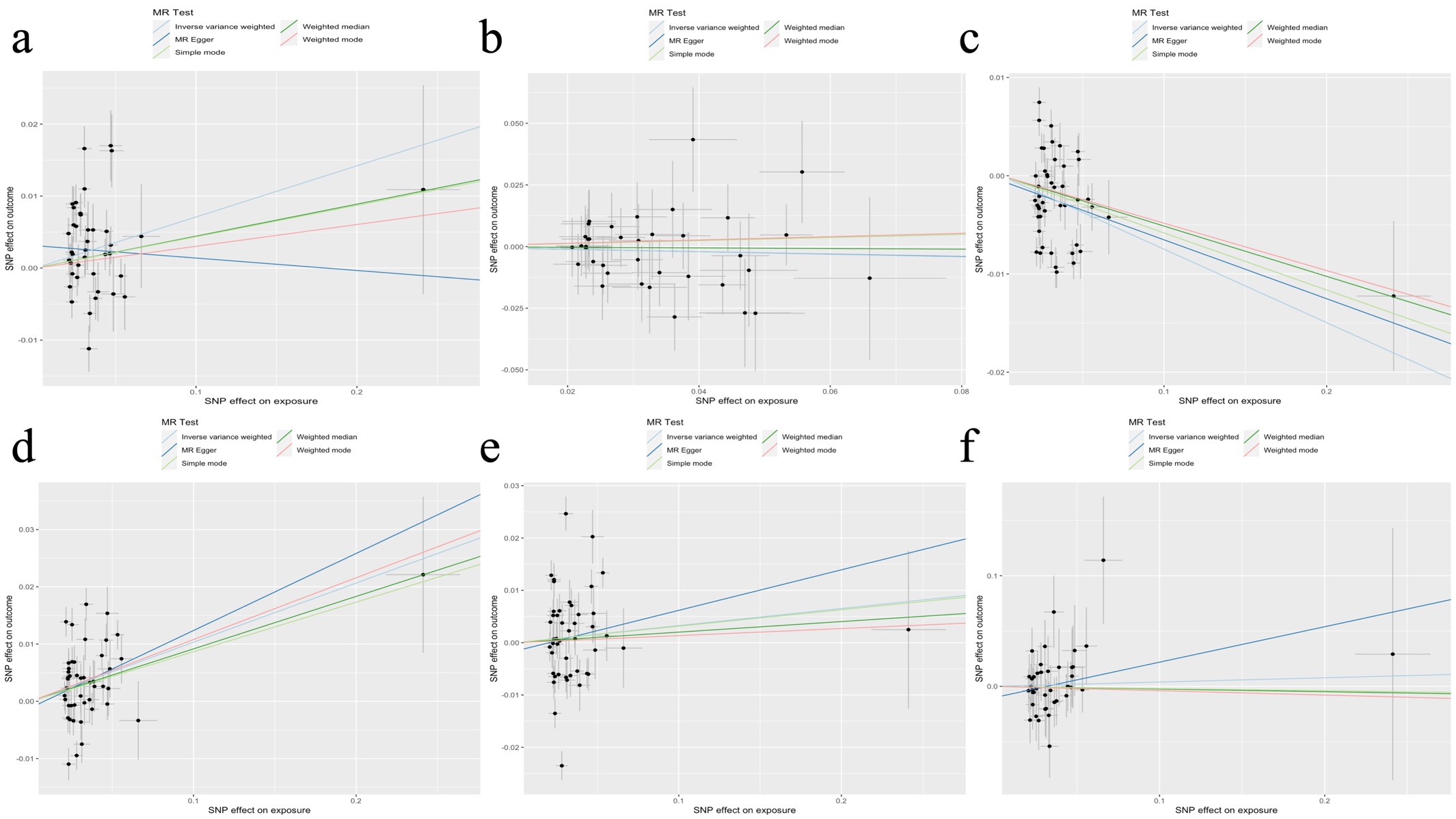
Supplementary Figure 3. The results of scatter plot.**


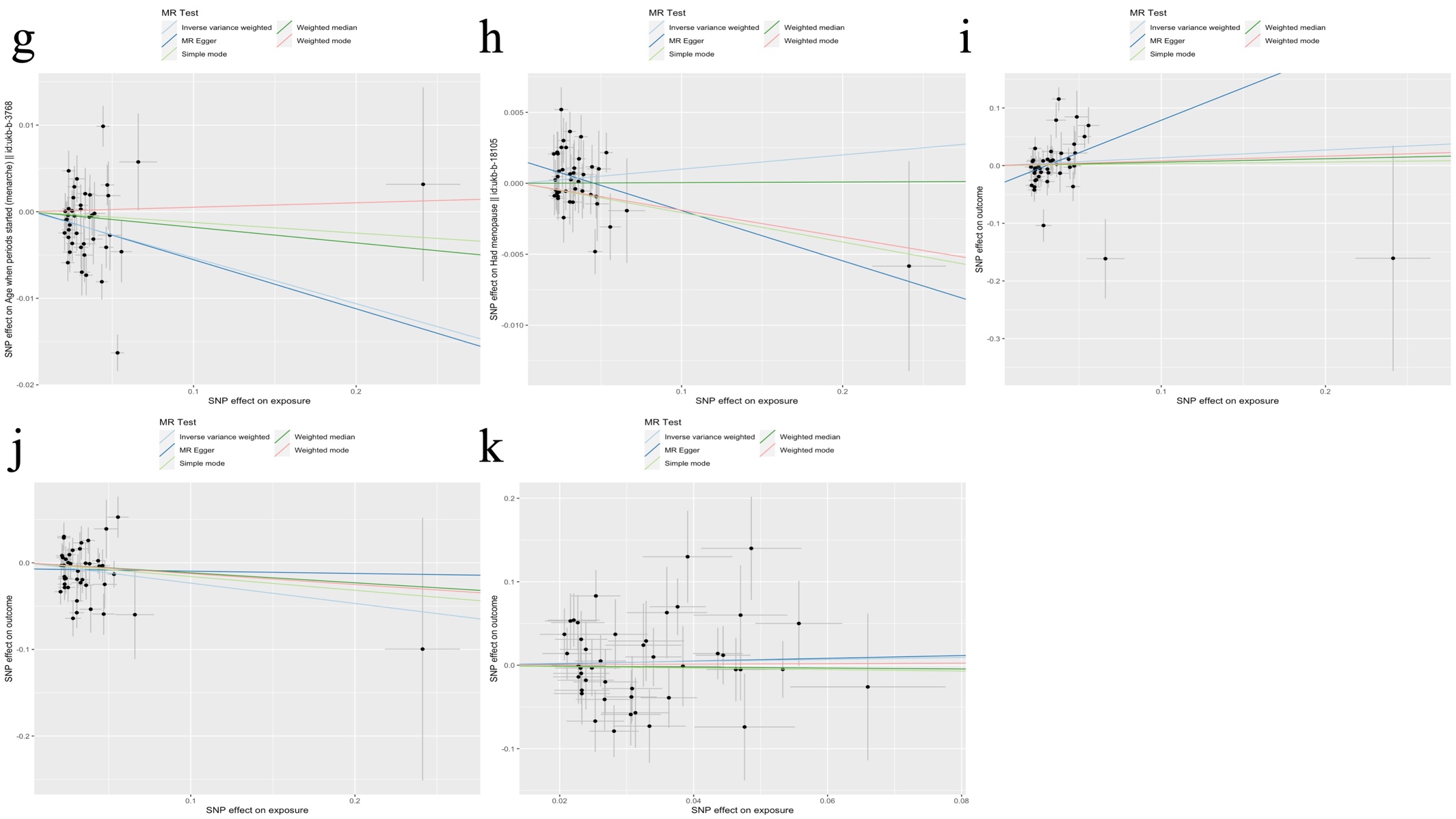
**Supplementary Figure 3. continued.**

**
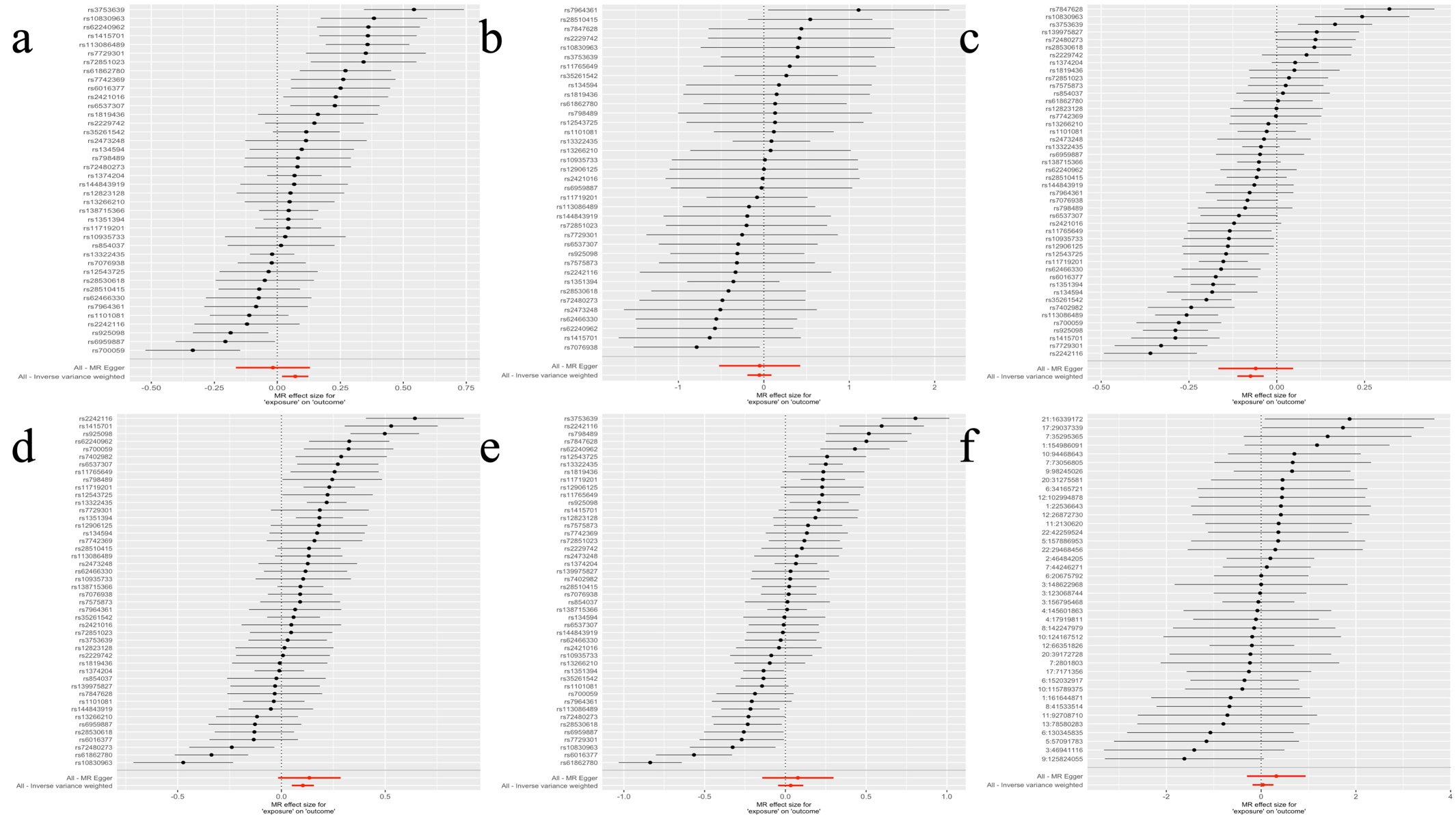
Supplementary Figure 4. The results of forest plot.**


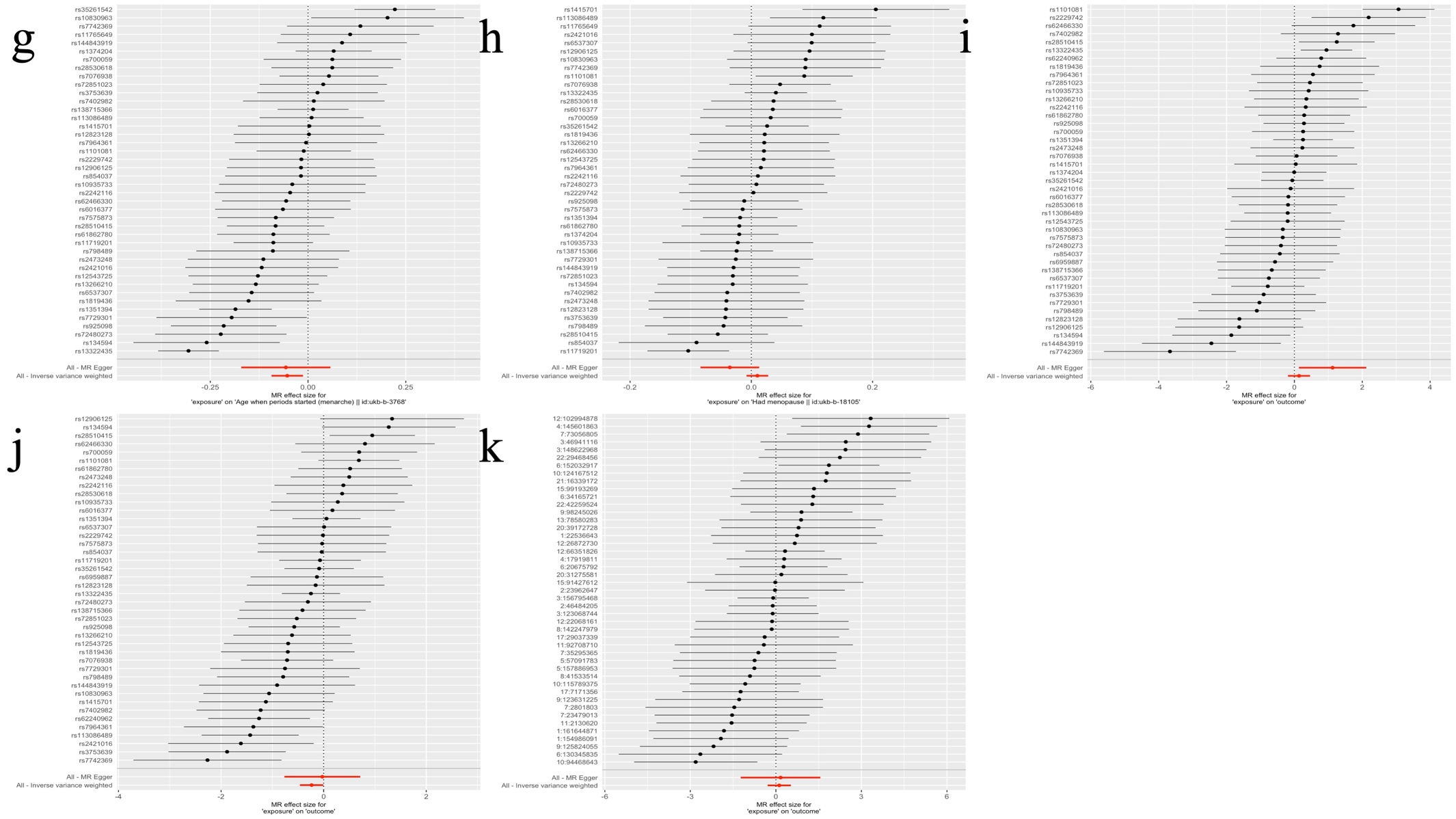
**Supplementary Figure 4. continued.**


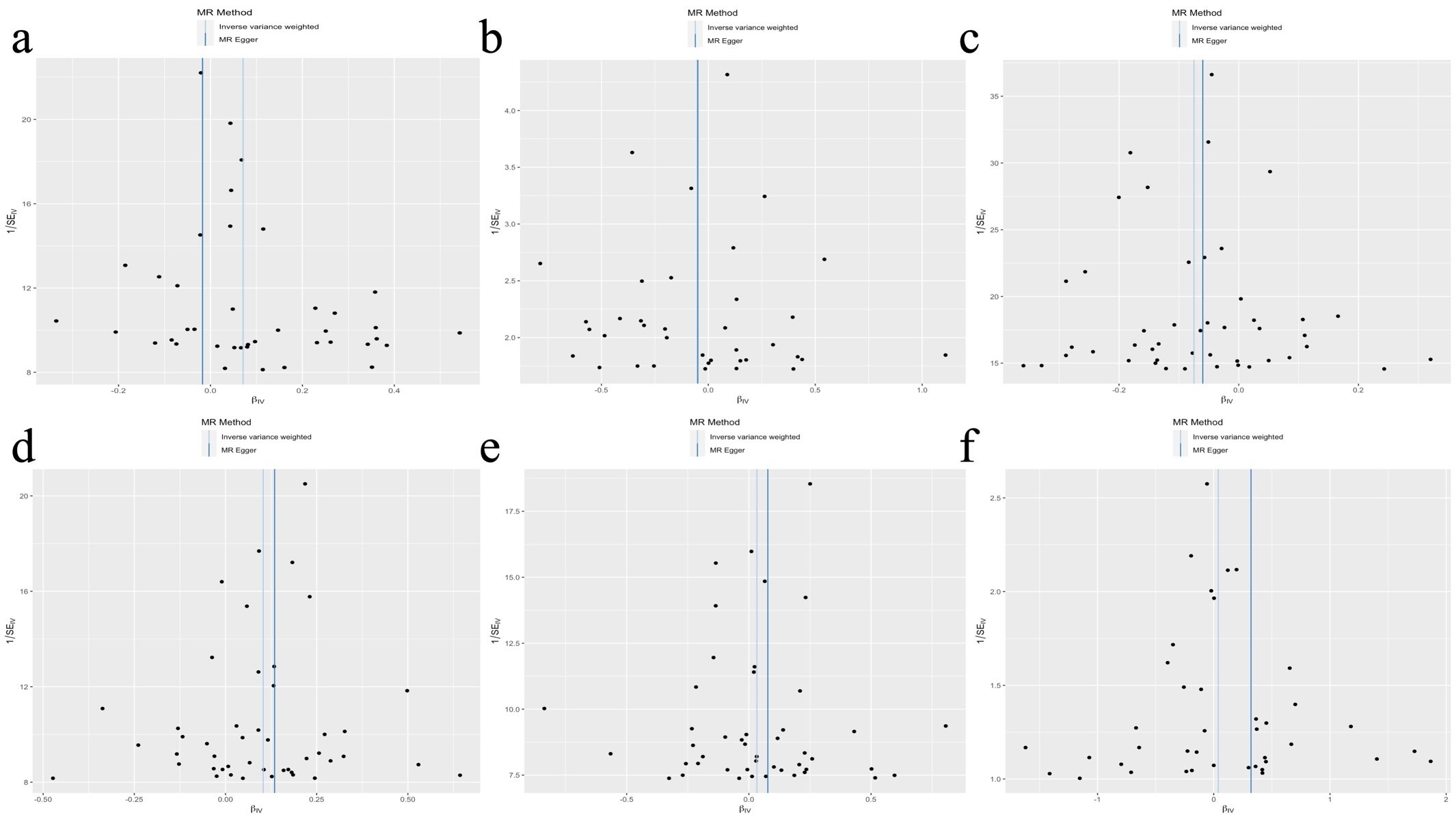
**Supplementary Figure 5. The results of funnel plot.**


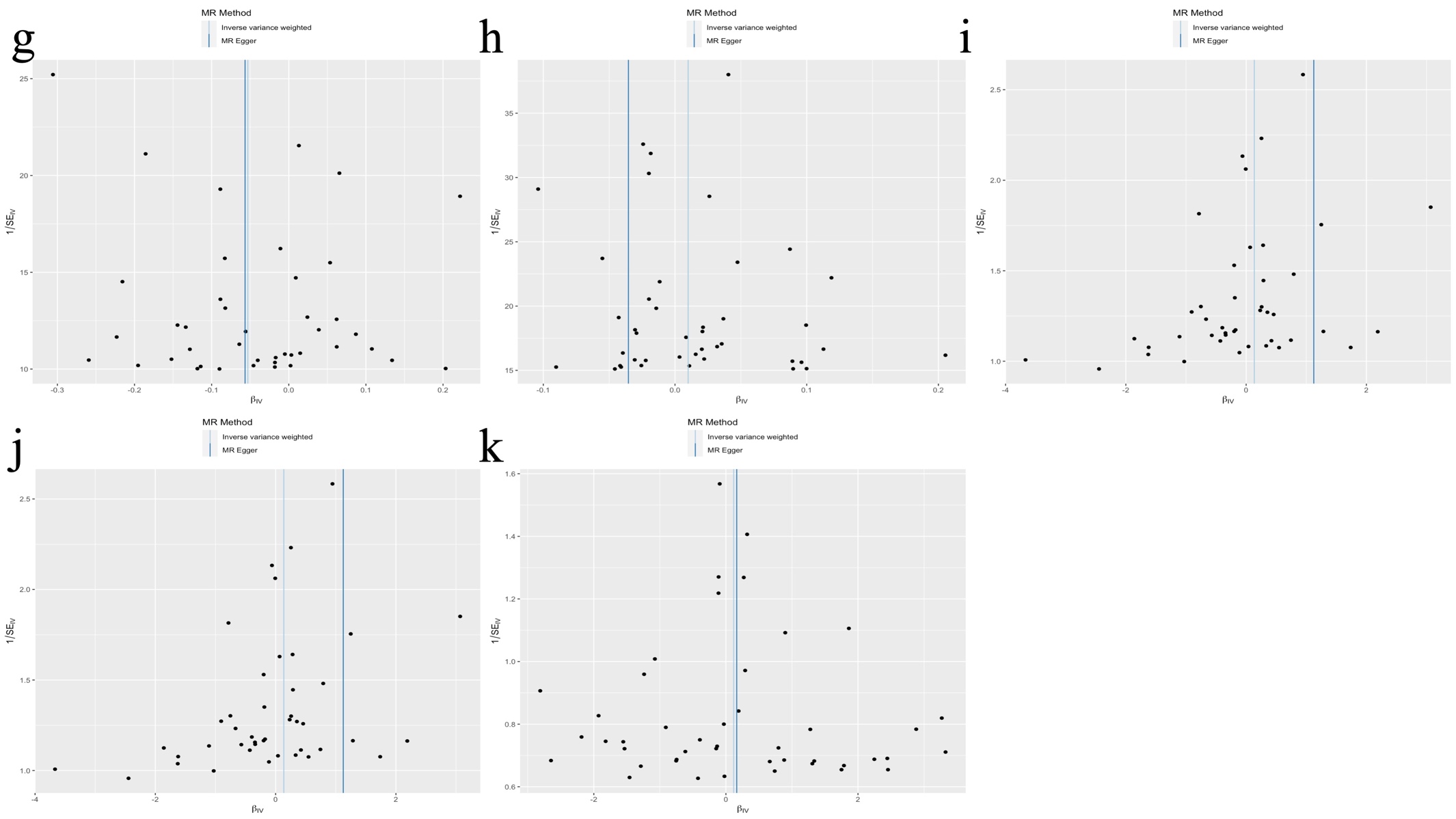
**Supplementary Figure 5. continued.**

1. **k**: **a**: body mass index, BMI; **b**: estradiol, E_2_; **c**: sex-hormone-binding globulin, SHBG; **d**: bioavailable testosterone, bio-T; **e**: total testosterone, TT; **f**: anti-mullerian hormone, AMH; **g**: menarche; **h**: menopause; **i**: endometriosis; **j**: leiomyoma; **k**: polycystic ovaries syndrome, PCOS)
